# Role of Dihydride and Dihydrogen Complexes in Hydrogen Evolution Reaction on Single-Atom Catalysts

**DOI:** 10.1021/jacs.1c10470

**Published:** 2021-11-25

**Authors:** Giovanni Di Liberto, Luis A. Cipriano, Gianfranco Pacchioni

**Affiliations:** Dipartimento di Scienza dei Materiali, Università di Milano-Bicocca, Via R. Cozzi 55, 20125 Milano, Italy

## Abstract

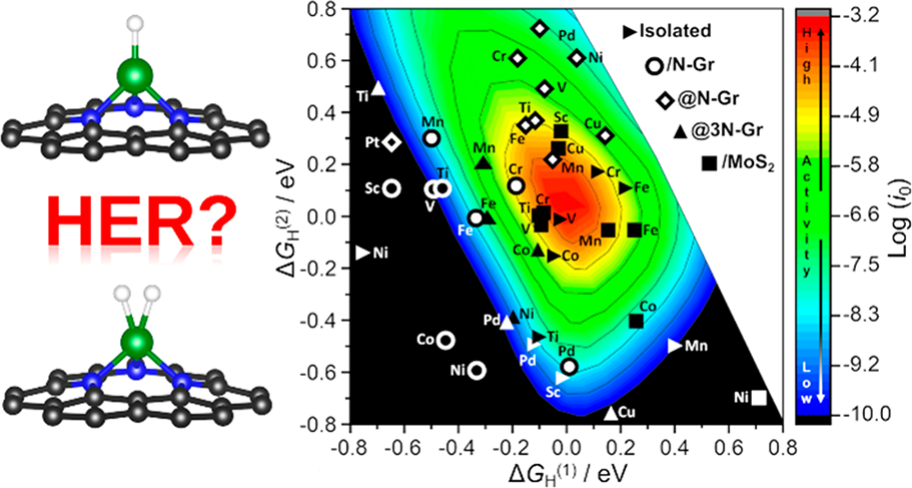

The hydrogen evolution reaction (HER) has a key role in electrochemical water splitting. Recently a lot of attention has been dedicated to HER from single atom catalysts (SACs). The activity of SACs in HER is usually rationalized or predicted using the original model proposed by Nørskov where the free energy of a H atom adsorbed on an extended metal surface M (formation of an MH intermediate) is used to explain the trends in the exchange current for HER. However, SACs differ substantially from metal surfaces and can be considered analogues of coordination compounds. In coordination chemistry, at variance with metal surfaces, stable dihydride or dihydrogen complexes (HMH) can form. We show that the same can occur on SACs and that the formation of stable HMH intermediates, in addition to the MH one, may change the kinetics of the process. Extending the original kinetic model to the case of two intermediates (MH and HMH), one obtains a three-dimensional volcano plot for the HER on SACs. DFT numerical simulations on 55 models demonstrate that the new kinetic model may lead to completely different conclusions about the activity of SACs in HER. The results are validated against selected experimental cases. The work provides an example of the important analogies between the chemistry of SACs and that of coordination compounds.

## Introduction

The hydrogen evolution reaction (HER) is one of the simplest but at the same time most important reactions in chemistry, 2 H^+^ + 2e^–^ → H_2_. It is a two-electron transfer reaction occurring at the cathode in electrochemical water splitting, H_2_O → H_2_ + ^1^/_2_O_2_.^1−4^ It offers the potential to produce H_2_, a critical chemical species, important both as a reagent (e.g., in ammonia synthesis)^5^ and as a fuel (e.g., in fuel cells technology)^6^ and in several other industrial processes (e.g., electronics, metallurgy, chemical industry). Today, a great interest is devoted to the HER using renewable sources of energy (solar, wind, geothermal, etc.), as this can open the way toward a sustainable source of hydrogen for transportation or industrial processes.^7−9^ To achieve a high energetic efficiency for water splitting, specific catalysts that minimize the overpotential necessary for the HER are needed.^10^ Among the best catalysts is platinum, which however has the disadvantage of being a critical and costly raw material.^11,12^ For this reason, an intense activity has been dedicated to the search for new materials with high catalytic efficiency for the HER.

Among new catalysts for the HER, there is the emerging class of single-atom catalysts (SACs).^13−15^ The concept of SAC was introduced in 2011,^16^ although the presence of isolated atoms or single sites at the surface of heterogeneous catalysts has been known for a longer time.^14,17−19^ SACs consist of isolated metal atoms dispersed and stabilized on a support, and their chemistry is largely determined by the interaction with the surrounding (in this respect, they are less “single” than the name suggests). Certainly, in terms of structure SACs are better defined than supported metal particles, where the activity is largely dependent on the size and shape of the nanoparticle.^20^ Since in SACs the active site is relatively well-defined, they are expected to provide higher selectivity than supported nanoparticles. SACs have the additional advantage of using tiny quantities of precious metals, thus making them interesting for the design of novel classes of industrial catalysts.

Theoretical studies based on electronic structure calculations, in particular on density functional theory (DFT), have proven useful for the preliminary screening of the large set of new catalytic materials for HER.^21−23^ The screening of the “best” catalysts for HER is usually done following the model proposed by Nørskov and co-workers in a seminal paper.^24^ This approach allows one to construct a volcano plot following the Sabatier principle, a qualitative concept in heterogeneous catalysis that states that the interactions between the catalyst and the substrate should be neither too strong nor too weak. Recently, this principle was revisited by including in the analysis the applied overpotential resulting in an extended Sabatier principle.^25−27^

Coming back to Nørskov’s model, the exchange current *i* for H_2_ evolution, a measure of the efficiency of the reaction, is plotted against the H atom adsorption free energy, Δ*G*_H_. This model, originally developed for H_2_ evolution from extended metal surfaces, is commonly employed in its original formulation also for the study of SACs.^28−35^ The basic assumption of this model is that the adsorption free energy of the H atom is the only parameter required, as the recombination of two H atoms on the active site gives rise to the formation and desorption of H_2_ into the gas phase. This hypothesis is widely verified, and in fact on metal surfaces H_2_ can exist only in dissociated form or in a physisorbed state where the molecule is weakly bound to the surface by van der Waals forces.^36^ However, the mechanism of H_2_ formation and release can be very different on SACs.

SACs can be considered surface analogues of organometallic complexes.^37−39^ In organometallic chemistry the existence and the nature of stable dihydride and dihydrogen complexes is well established.^40,41^ These consist of two H atoms bound to the same metal center. Changing the ligands, L_*x*_, the level of activation and population of the H_2_ antibonding orbital can be tuned, with formation of a classical dihydride complex, ML_*x*_(H)(H) (oxidative addition), or of the less conventional dihydrogen complexes ML_*x*_(H_2_) where the H–H bond is only partly activated (typical H–H distance 0.8–0.9 Å). Pioneering work by Kubas has shown that dihydrogen complexes can have high chemical stability.^42,43^ Since SACs have clear analogies with coordination complexes, it is to be expected that these kinds of HMH intermediates can also form in this case. *When this occurs, the original computational recipe of HER on metal surfaces can be insufficient, and a more elaborated model that accounts for the formation of two-hydrogen complexes is required*. This does not necessarily imply that the original model leads to incorrect conclusions: as we will show below, only if both H adsorption processes are relevant does this affect the prediction of the kinetics of the reaction. However, it is not possible to exclude a priori the existence of the HMH intermediates and their role in the reaction.

In this work, we provide an extension of the original Nørskov’s theory by developing a theoretical approach that accounts for the presence of two intermediates in HER on SACs, the single hydrogen, MH, and the two-hydrogen, HMH, complexes. We do not include explicitly the effect of the overpotential and of the solvent, as it will be discussed below, since these are not included in the original model as well. However, we will show that neglecting the possible formation of the second intermediate can have much larger effects.

We provide the fundamental kinetic equations and report numerical DFT simulations demonstrating that (a) the formation of the two intermediates is possible for most of the SACs considered, (b) the HMH intermediate can have different natures, dihydride or dihydrogen, depending on the interaction with the support, and (c) the proposed model should be used when predicting the activity of SACs, since the original model, designed and well-grounded for metal surfaces, may lead to different conclusions about the activity of SACs in HER. The model proposed here, validated for a selected number of experimental SACs, is general and can be applied for a preliminary screening of the HER activity of any catalyst made by supported single atoms, as well as of homogeneous catalysts based on coordination compounds.

## Results and Discussion

### Modeling of HER on Metal Surfaces

Before we present the new kinetic model for HER on SACs, we briefly summarize the basic features of the original model. In standard conditions, the change of Gibbs free energy, Δ*G*^0^, for the semireaction 2H^+^ + 2e^–^ → H_2_ is equal to zero. Thus, the potential needed to promote the reaction is also zero, since Δ*G*^0^ = −*nFE*^0^ (*n* = the number of electrons involved, *F* = Faraday’s constant, *E*^0^ = the reduction potential). However, in catalytic processes this is true only in principle, since an extra potential, the overpotential η, is required for the reaction to occur; in the original model, it is assumed that η = 0, an approximation that has been the subject of intense work in the past few years, which leads to a small correction.^25−27^ When a proton is reduced on a metal catalyst, the reaction is
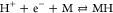
1where M is a metal site and MH denotes an adsorbed H atom on the surface. In this step, the Volmer step, Δ*G*^0^_H_ can differ from zero. In particular, if the H atom is strongly bound to the surface Δ*G*^0^_H_ < 0; vice versa, if H is weakly bound, then Δ*G*^0^_H_ > 0. Now, in order to form H_2_ the reaction can follow two different paths. The first mechanism, the Heyrovsky reaction, involves the direct reduction of the second proton on the same metal site, i.e.,
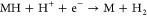
2

In the second path, the Tafel reaction, two H atoms bound to two different metal sites can combine:
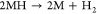
3

Usually the two paths occur in parallel, i.e., both are relevant, and their predominance depends on the H coverage.^44,45^ Once the H_2_ molecule forms, it can weakly bind to the surface thanks to dispersion forces. However, the physisorbed intermediate is almost at the same energy of the separated systems, M + H_2_,^46,47^ and it is fully justified to ignore this minimum in the overall thermochemistry of the reaction. This also means that the overpotential can be simply described by Δ*G*^0^_H_, either following Volmer–Heyrovsky (VH) or Volmer–Tafel (VT) paths. This behavior was described by Trasatti making use of a volcano curve where the exchange current, *i*_0_, of HER on different metal catalysts is plotted against the strength of the M–H bond (the exchange current is proportional to the amount of H_2_ produced).^48^

In their seminal paper, Nørskov and co-workers provided a link between the empirical observations and the atomistic calculation of the H adsorption free energy under the basic assumption that every reaction barrier other than thermochemistry is neglected.^24^ The approach consists in evaluating the adsorption energy of an H atom on a metal (Δ*E*_H_) computed with respect to the clean surface M and gas-phase H_2_:
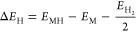
4

**Figure 1:**
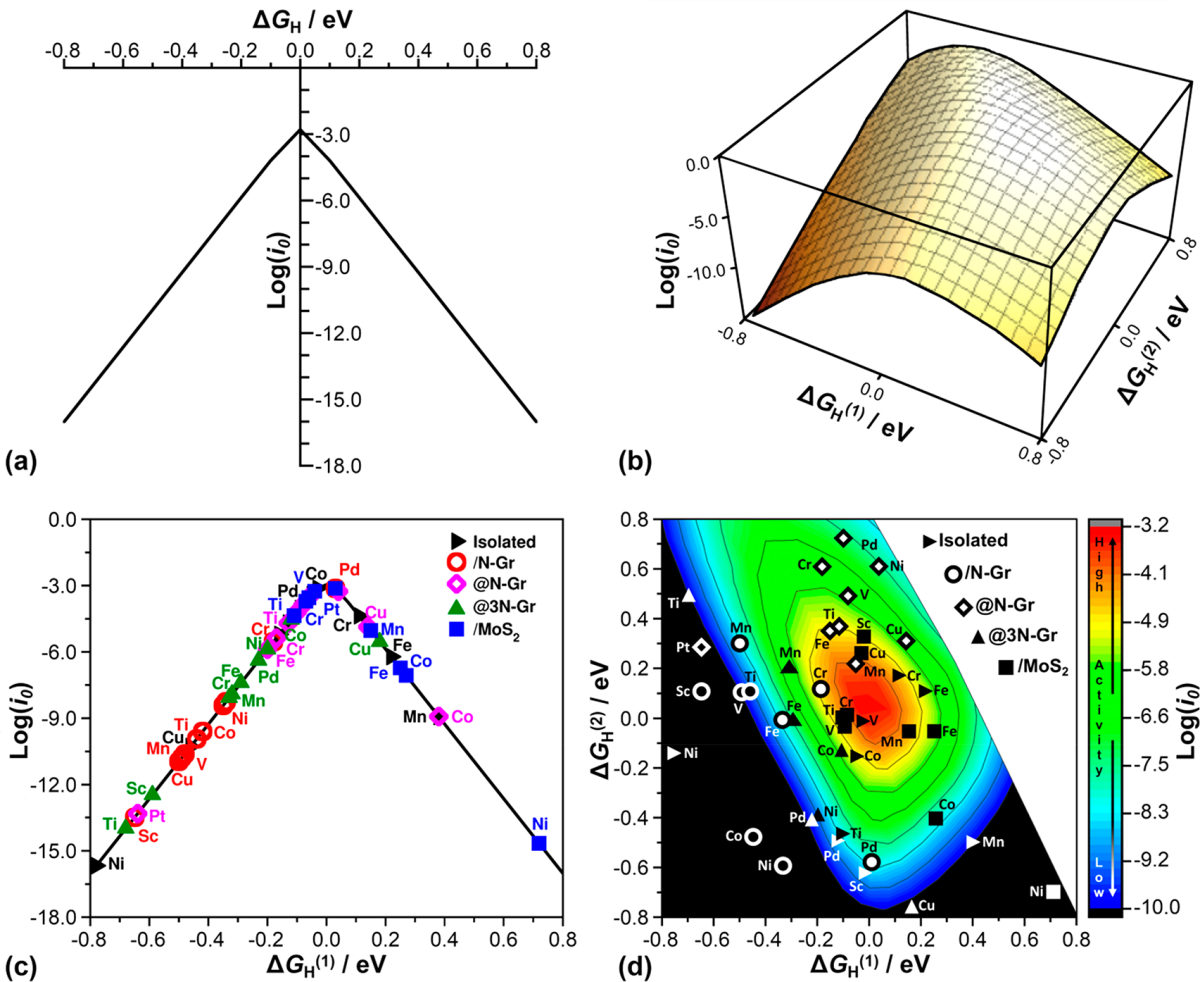
(a) Two-dimensional volcano plot for HER reaction assuming the formation of an MH intermediate (classical kinetic model); (b) three-dimensional volcano plot for HER reaction assuming the formation of MH and HMH intermediates (new kinetic model proposed here); (c) two-dimensional volcano plot derived from numerical DFT results for the case of HER reaction on 55 SACs computed assuming the formation of a single MH intermediate; (d) three-dimensional volcano plot derived from numerical DFT results for the case of HER reaction on 55 SACs assuming the formation of MH and HMH intermediates. Red: high activity; blue: low activity. When log(*i*_0_) < −10 (extremely low activity), the color is black. Intermediates having both Δ*G*_H_^(1)^ and Δ*G*_H_^(2)^ between −0.8 and 0.8 eV are reported in panels (c) and (d).

Then, it is possible to calculate the change of Gibbs free energy as
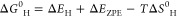
5Here, Δ*E*_ZPE_ is the zero-point energy correction, and Δ*S*^0^_H_ is the entropy variation of the reaction at standard conditions, usually approximated as . Δ*G*^0^_H_ varies with the H coverage,^49^ but this dependence is usually neglected. Irrespective of the VH or VT paths for H_2_ evolution, the relation between exchange current *i*_0_ and Δ*G*^0^_H_ can be represented as a volcano plot, Figure 1(a). This can be analytically derived by solving the kinetic equations under the steady-state approximation for the MH reaction intermediate (see Supporting Information).^50^ For simplicity, the equations are reported at pH = 0. This leads to two working equations for VH (eq 6) and VT (eq 7) paths, at standard conditions:
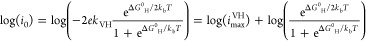
6
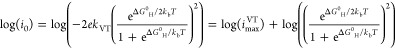
7*k*_VH_ and *k*_VT_ are the kinetic constants of the VH and VT paths, respectively. The top of the volcano plot, Figure 1(a), corresponds to Δ*G*^0^_H_ = 0; the left side of the plot is associated with metal surfaces that bind H too strongly, the right side to metals that bind H too weakly. The best condition consists in a metal that binds H neither too strongly nor too weakly, as in this case the required overpotential η tends to zero, making the reaction favorable. This means that the prediction of the activity of any metal catalyst in HER can be deduced simply from the adsorption energy of a H atom on that metal surface, i.e., by evaluating Δ*G*^0^_H_, hence the overpotential η. This strategy has been widely and successfully applied in several computational studies of HER on metal surfaces.^51−53^

### Modeling of HER on Single-Atom Catalysts

The approach described above, derived for an extended metal surface, has been applied so far in its original formulation also to the case of SACs. However, SACs, isolated metal atoms anchored on a support, can behave quite differently from an atom of a metal surface. As we mentioned in the Introduction, transition metal atoms surrounded by ligands are at the core of coordination chemistry, and several examples have been reported where a single transition metal (TM) atom can bind and coordinate two H atoms, forming HMH complexes.^40−43,54,55^ In this respect, the Volmer step on SACs, i.e., the formation of the MH surface complex, eq 1, can be followed by the addition of a second H atom with the formation of a stable HMH intermediate. This introduces a new step in the process, with the consequence that the kinetic equations describing the evolution of H_2_ become considerably more complex. The exchange current in this case depends on two variables instead of one:

(i) the free energy change for the adsorption of the first H atom (Δ*G*^0^_*H*^(1)^_), as in the conventional kinetics of metal surfaces:
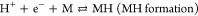
8
(ii) the free energy change for the adsorption of the second H atom (Δ*G*^0^_H^(2)^_):

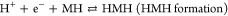
9

Furthermore, the third reaction step, H_2_ evolution,
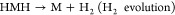
10is also a function of Δ*G*^0^_H^(1)^_ and Δ*G*^0^_H^(2)^_, because the overall reaction 2H^+^ + 2e^–^ → H_2_ is thermoneutral in standard conditions. This leads to
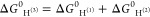
11

Solving the kinetic equations using the same approximations done for the case of metal surfaces, one obtains the following expression for the exchange current *i*_0_ (Supporting Information, Kinetics of HER):
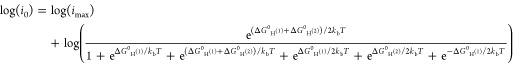
12

Interestingly, the exchange current is still described by a volcano plot, Figure 1(b), where the maximum current occurs when both Δ*G*^0^_*H*^(1)^_ and Δ*G*^0^_*H*^(2)^_ are close to zero; that is, the formation of H adsorbates is thermoneutral. In this respect, the key requirements for a good HER catalyst are unchanged. However, in the case of HER on SACs two formation energies, and not just Δ*G*^0^_*H*^(1)^_, determine the overall performance of the catalyst.

Of course, as we mentioned above, if the rate-determining step of the reaction is the formation of the first intermediate, MH, one recovers the typical two-dimensional volcano plot of the standard model, Figure 1(a). However, if the second intermediate is sufficiently stable, the overall kinetics cannot be described by one variable. So far, this possibility has not been considered. Below we will provide some numerical examples based on first-principles calculations of the different conclusions that can be reached if one neglects the formation of the HMH intermediate using the classical one-variable model, and we report some benchmark of the proposed model against available experimental measurements.

### Experimental Benchmark

We first provide a validation of the proposed kinetic model by comparing the predicted behavior with experiment for a few selected cases. This kind of comparison is far from simple. First of all, the results of the DFT calculations can be compared to experiment only if the structure of the active SAC is the same. While the structure is defined at an atomistic level in the calculations, this is not always the case in experiment. Electron microscopy, X-ray spectroscopies (XANES and EXAFS), high-resolution STEM, infrared spectroscopy of adsorbed probe molecules, etc., often in combination, can provide essential information and sometimes unambiguous identification of the structure of the catalyst.^56^ More often, the nature of the SAC is only partly elucidated, making a direct comparison with DFT problematic. The second aspect is the dynamical behavior of SACs under reactive conditions.^57,58^ The local coordination and position of a SAC can change in the course of the reaction depending on the oxidizing or reducing environmental conditions.^59^ A third problem is that often the catalyst contains small aggregates beside SACs, and the overall activity can be due to a combination of active sites. A fourth problem is that DFT total energies are affected by intrinsic errors,^60^ as reaction free energies depend on the details of the calculation (exchange–correlation functional, size of the supercell, nature of the pseudopotential, treatment of dispersion, etc.). Finally, we have seen above that recent work has shown the importance of other effects like the applied overpotential^25−27^ and the interaction with the solvent.^61^ Taking into account all these aspects, we have identified four representative experimental studies for a total of six catalysts where the nature of the SAC has been clearly identified, so that a comparison with our DFT calculations is meaningful.

The comparison is based on a key physical quantity in HER, the overpotential η, as the absolute value of the exchange current, *i*_0_, depends on a kinetic constant which is unknown. The overpotential η coincides with the absolute value of free energy of the reaction, |Δ*G*_H_|; if the reaction occurs in one step, η = |Δ*G*_H_^(1)^|/*e*; if two steps are involved, MH and HMH intermediates, η = |Δ*G*_H_^(1)^ + Δ*G*_H_^(2)^|/*n*e, where *n* is the number of electrons involved (two in this case).

**Figure 2:**
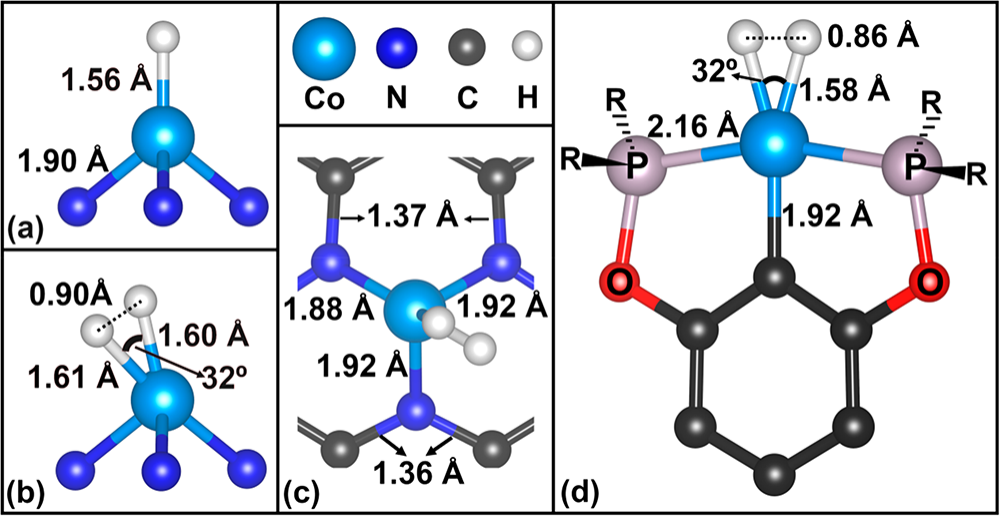
(a) Side view of CoH@3N-Gr intermediate; (b) side and (c) top views of the HCoH@3N-Gr dihydrogen intermediate (DFT calculations); in (a) and (b) the (3N-Gr) support is not reported for clarity. (d) Side view of a Co dihydrogen coordination compound isolated experimentally; the geometry reported has been obtained from DFT calculations of the new complex.^66^ Selected distances and angles are reported.

As an example of the computational procedure followed (see Supporting Information: Computational Details), we consider the case of a Co atom stabilized at N-doped graphene, Co@3N-Gr (Supporting Information, Figure S1), a system that has been studied both theoretically and experimentally.^33,62,63^Figure 2(a–c) report the structures of the CoH@3N-Gr and HCoH@3N-Gr complexes, respectively. The H---H distance in HCoH is 0.90 Å, indicating the formation of an activated H_2_ molecule, a precursor state of H_2_ desorption.^64,65^ Both calculated H–H (0.90 Å) and Co–H (1.56 Å) bond lengths for the Co@3N-Gr SAC compare very well with those reported for an experimentally isolated Co dihydrogen complex (0.86 and 1.57 Å, respectively, derived from DFT calculations^66^), Figure 2(d).

It is interesting to compare the predicted HER activity for Co@3N-Gr with the experimental results of Fei et al., who reported a high activity for this catalyst.^62,67^ For each path, one or two intermediates, we have calculated the exchange current and the Δ*G*_H_ values using the equations reported above. The measured overpotential (0.15–0.18 eV)^68^ is well reproduced using both a kinetic model based on a single intermediate (Δ*G*^0^_H^(1)^_ = −0.12 eV) or on two intermediates (Δ*G*^0^_H^(1)^_ = −0.12 eV, Δ*G*^0^_H^(2)^_ = −0.15 eV) (Supporting, Table S1). Thus, this is a case where the formation of the dihydrogen complex does not alter the conclusions obtained with the original model since both steps have Δ*G*^0^ ≈ 0. However, this is not a representative example, and in other cases quite different results are obtained with the two procedures.

The second case is that of Co atoms supported on MoS_2_, Co/MoS_2_. Usually, TM atoms are incorporated into the lattice of MoS_2_ replacing a Mo cation;^69,70^ when this occurs, the activity of the surface S atoms near the TM ion changes and results in good HER activity (rather than SACs, these systems should be better classified as doped MoS_2_). Recently, a system consisting of Co atoms supported on the surface of MoS_2_ has been reported and an overpotential η = 84 mV for the HER has been measured at 10 mA cm^–2^.^71^ High-angle annular dark-field STEM (HADDF-STEM) images indicate that the Co atoms adsorb on the Mo-top sites; thus this is the site considered in the calculations. If we use the classical model, Δ*G*_H_^(1)^ = 340 meV (η = 340 mV); when we consider also the second step, Δ*G*_H_^(2)^ = −665 meV, we have Δ*G*_H_^(3)^ = −324 eV and η = |Δ*G*_H_|/2 = 162 mV (Supporting Figure S2 and Table S2). This is consistent with Co/MoS_2_ being a catalyst with a very low overpotential for HER. Notice that if we assume that the Co atom is on the hollow site of MoS_2_ instead of on top of Mo, the corresponding overpotentials are η = 269 mV (one step) and η = 59 mV (two steps): also in this case Co is predicted to be an efficient catalyst and the agreement with experiment is quantitative (but possibly fortuitous).

The third example deals with Ni atoms trapped at defective graphene, Ni@D-Gr. In this atomically dispersed catalyst, Ni atoms are stabilized at a C-divacancy where they are coordinated to four C atoms in a square planar geometry, as shown HADDF-STEM and XANES measurements.^72^ The catalyst exhibits an overpotential of 70 mV at 10 mA cm^–2^. Using a model of Ni@D-Gr (Supporting Figure S3 and Table S3), we computed η = 464 mV with the classical model and η = 197 mV with the two-step model. While the two-H model is more or less consistent with experiment (197 mV versus 70 mV), the single-step model is completely inconsistent (464 mV versus 70 mV).

The final example deals with M atoms stabilized at N-doped graphene. In a recent study three catalysts where Co, Ni, and W atoms occupy a C-divacancy and are coordinated to 4 N atoms have been synthesized and characterized, M@4N-Gr.^33^ The three catalysts have been selected based on DFT calculations that showed that Co@4N-Gr is expected to be much more active in HER than Ni@4N-Gr and W@4N-Gr. Using the classical model, in fact, the DFT overpotentials predicted in ref (33) are 130 mV for Co and 930 mV and 1620 mV for W and Ni.^33^ According to these values, only Co@4N-Gr should be active, while W@4N-Gr and in particular Ni@4N-Gr should be totally inert. The measured overpotentials at 10 mA cm^–2^ however are 230 mV (Co), 530 mV (W), and 590 mV (Ni),^33^ showing that all three catalysts may be active, with Co exhibiting the lowest overpotential. We have computed η for Co@4N-Gr, Ni@4N-Gr, and W@4N-Gr catalysts with the two kinetic models (Supporting Figures S4–S6 and Table S4). With the classical model we obtain η = 162 mV (Co), η = 882 mV (W), and η = 1614 mV (Ni), the same DFT values reported in ref (33). However, when also the HMH complex is considered, the computed overpotentials are 257 mV for Co and 439 mV for W, in quantitative agreement with the experiment (230 and 530 meV, respectively).^33^ Quite interesting is the case of Ni, since here neither dihydride nor dihydrogen complexes form. The adsorption of a second H leads to a H_2_ molecule weakly physisorbed (Supporting Figure S6). This is a typical case where the conventional one-step model is valid. This also means that the computed overpotential for Ni is η = 1614 mV, in agreement with the theoretical value reported in ref (33), but in contrast with the measured overpotential of 590 mV. We can only speculate that the structure of the Ni catalyst is probably different from that considered here, but more work is required to clarify this point.

Overall, the new model accounts for the features observed experimentally and provide a better agreement with the experimental data than the classical one-step model. The results have been obtained using the PBE exchange–correlation functional. In order to assess the importance of this choice on the final results, the analysis for M@4N-Gr has been repeated using the PBESOL and revPBE functionals (see Supporting Figure S7 and Tables S5 and S6). While differences of up to 250 meV are found in the adsorption energies, the same general conclusions are obtained with the three functionals: the HMH model improves the agreement with experiments for Co@4N-Gr and W@4N-Gr, while for Ni@4N-Gr no model (MH or HMH) provides a good result, suggesting that the Ni catalyst occupies a different site.

### Formation of Two-Hydrogen Complexes on SACs

To assess the role of two-hydrogen HMH complexes in the prediction of the catalytic activity of SACs, we have performed DFT calculations on a larger series of model systems: (a) isolated, gas-phase TM atoms (from Sc to Cu, Pd, and Pt); the same group of atoms adsorbed on N-doped graphene, M/N-Gr (b); replacing a C atom in N-doped graphene, M@N-Gr (c); replacing a C atom in graphene doped by three N atoms (pyridinic), M@3N-Gr (d); and M adsorbed on MoS_2_, M/MoS_2_ (e). In this way we generated a hypothetical set of 55 systems to model the HER on SACs. For each system we have considered the activity as predicted (I) by the classical one-intermediate model and (II) by the new two-intermediates model (formation of HMH complexes).

The results are reported in Supporting Tables S1, S7–S15 and Figures S8–S22. In most cases the adsorption of two H atoms on the 55 models of SACs results in the formation either of dihydride or of dihydrogen complexes. In particular, all the isolated TM atoms considered lead to the classical dihydride species, characterized by long H---H distances (from 1.75 to 3.25 Å) and short M–H distances (Supporting Table S7 and Figure S8), consistent with matrix-isolation experiments.^73^ For M atoms adsorbed on N-Gr, M/N-Gr, only Co, Ni, and Pd form dihydrogen complexes; the rest give rise to dihydride species (Supporting Table S10 and Figure S11). When the metal atoms are incorporated in N-Gr, M@N-Gr, we found four cases of dihydrogen species (Ti, Cr, Mn, Fe), the rest being dihydrides (Supporting Table S12 and Figure S15); for M@3N-Gr the dihydrogen complexes form in the case of Mn, Fe, Co, Ni, and Cu (Supporting, Table S1 and Figure S19), while when the metal atoms are supported on MoS_2_, the majority of SACs form dihydrogen species (Ti, V, Cr, Mn, Fe, Co, Ni, Cu, and Pd) (Supporting Table S15 and Figure S22).

The reasons for the formation of dihydride or dihydrogen complexes have been widely discussed for organometallic complexes and depend on the nature of the ligands (electron donor species favor dihydride, electron attractor ligands favor dihydrogen). This is apparent if one compares free, unsupported, and MoS_2_-supported transition metal atoms: the free metal atoms have sufficient electron density to break the H_2_ molecule so that only dihydride complexes are formed; on MoS_2_ the bonding of M with three electronegative S atoms reduces the electron density and the dihydrogen complexes dominate. An efficient descriptor is the d-band center; for the case of a single H atom adsorption on M/MoS_2_ it has been shown that a linear correlation exists between the position of the d_*z*^2^_ orbital of M and the strength of the M–H bond.^74^

One might wonder why, if dihydride or dihydrogen complexes on SACs are predicted to exist by DFT calculations, they have not been observed. Actually, a few examples of metal atoms on surfaces binding two H atoms are known, although their relevance for HER has not been recognized so far. Well-documented cases of a transition metal atom anchored on a silica surface that binds more than one H atom have been reported by Basset and co-workers, [≡SiO)_2_ZrH_2_], [≡SiO)WH_3_(Me)_2_], and [≡SiO)_2_TaH_3_].^75−78^ Recently, combining inelastic neutron scattering and infrared spectroscopy, the formation of a dihydrogen complex has been demonstrated for Cu ions coordinated to a zeolite framework.^79^ The coexistence of dihydride and dihydrogen species has been reported for the Ru atoms of the RuO_2_(110) surface based on HREELS measurements and DFT calculations.^80^

The formation of dihydrogen and multihydrogen complexes has been observed in DFT calculations on the HER of Pt atoms on N-Gr,^13^ MoS_2_,^81^ onion-like carbon nanospheres,^82^ defective graphene, and h-BN,^83^ although their relevance for the kinetics of the process has not been fully appreciated. More difficult is the observation of HMH complexes on SACs stabilized on metal oxides, as here a tendency toward the spillover of one of the H atoms to the surface, with formation of MH and OH species, has been reported.^84,85^ Of course, the fact that HMH species can form on SACs does not necessarily means that these can also be observed in real samples at room temperature. In Supporting Tables S1, S7, S10, S12, and S15 we report the energy required to desorb H_2_ from the HMH complexes (this is the reverse of the H_2_ adsorption energy). The majority of cases studied show a positive H_2_ desorption energy, i.e., an endothermic process, but this ranges from a few tens eV for many complexes up to 2.86 eV for the case of HPtH@3N-Gr (Supporting Table S1). Using a Redhead equation for first-order desorption processes,^86^ Δ*E*_des_ = *RT*_des_ [ln(ν*T*_des_) – 3.64], and assuming a prefactor ν = 10^13^ s^–1^, one can estimate that thermal desorption of H_2_ from HMH is possible for temperatures above 300 K only if Δ*E* > 0.8 eV. This condition is fulfilled for a few cases: for M/N-Gr, Sc, Co, Ni, Pd, and Pt (Supporting Table S10); for M@N-Gr and M/MoS_2_ only Pt (Supporting Tables S12 and S15); for M@3N-Gr, V, Ni, Cu, Pd, and Pt (Supporting Table S1). When stable dihydrogen or dihydride complexes form, they can be detected using neutron diffraction or spectroscopic techniques such as ^1^H NMR and IR (Supporting Figure S26).^54^

### Comparison of Kinetics Based on One or Two Intermediates

Having discussed the formation, nature, and stability of the HMH complexes for the 55 SAC models, we consider now their impact on the kinetics of HER. Using the original Nørskov’s model, the plot of the exchange current vs Δ*G*_H_^(1)^ results in the classical two-dimensional volcano plot; see Figure 1(c). On the other hand, if one considers the adsorption of two H atoms on the catalytic center, the exchange current is plotted against Δ*G*_H_^(1)^ and Δ*G*_H_^(2)^, resulting in a three-dimensional volcano plot; see Figure 1(d).

**Figure 3:**
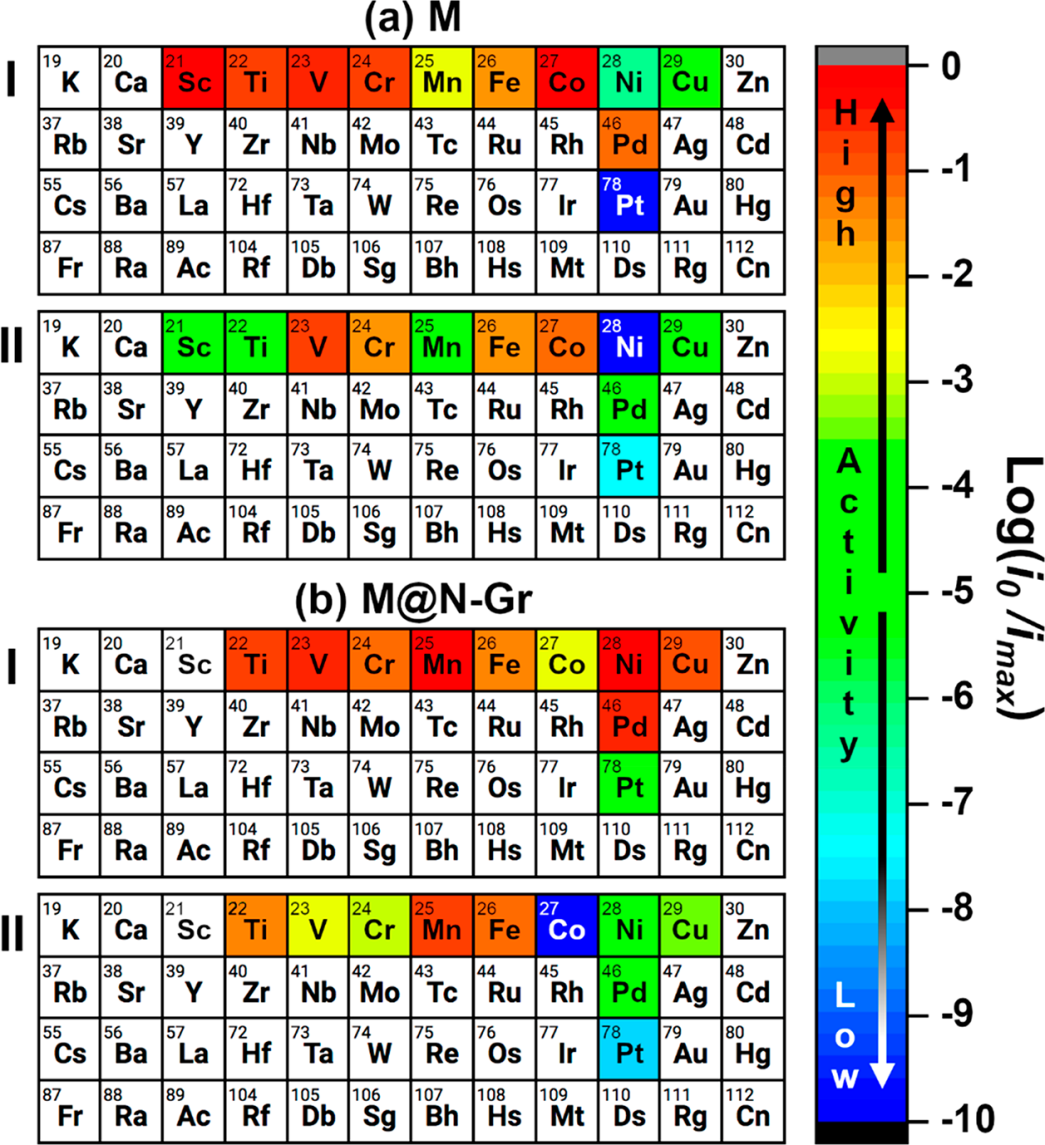
Predicted efficiency for the HER reaction for 22 models of SACs. (a) Free M atoms, M; and (b) M atoms incorporated on N-Gr, M@N-Gr. (I) Classical model based on a single MH intermediate; (II) model based on both MH and HMH intermediates (two-hydrogen complex). The color refers to the ratio of the exchange current *i*_0_ for a given atom and the maximum exchange current, *i*_max_, and provides a visual measure of the expected HER activity. The best catalysts (in red) have log(*i*_0_/*i*_max_) close to zero; the worse catalysts (in blue) have log(*i*_0_/*i*_max_) close to −10. Atoms are colored in black when log(*i*_0_/*i*_max_) < −10. On Sc@N-Gr the HMH complex does not form; only the one-intermediate model can be applied.

Both models lead to a volcano-type behavior, but the two volcano plots cannot be directly compared, being described by a different number of variables. Thus, we introduce a common descriptor of the efficiency of a given catalyst, defined as the ratio of the exchange current, *i*_0_, and the maximum exchange current, *i*_max_ (corresponding to the top of volcano curve). If the logarithm of *i*_0_/*i*_max_ is close to zero, then the SAC has an exchange current close to the top of the volcano curve, hence maximum efficiency. On the contrary, when the exchange current is low, log(*i*_0_/*i*_max_) is a negative number. In this way, we can directly compare the efficiency predicted by the two kinetic models. In Figure 3 we report a periodic table where the atoms investigated are color coded: red corresponds to the most active SACs, blue or black corresponds to the most inactive ones; green corresponds to a weak activity. The results are shown for isolated metal atoms M and for the same atoms stabilized at N-Gr, M@N-Gr (the other systems considered are reported in Supporting Figure S27).

Figure 3(a,I) refers to the predicted activity based on the classical MH intermediate model for isolated metal atoms; the same for the two-intermediate models is shown in Figure 3(a,II) (Supporting Table S7). The inclusion of the HMH intermediate in the kinetics leads to a drastic reduction in the number of “good” catalysts: with model I Sc, Ti, V, Cr, Mn, Fe, Co, and Pd are predicted to be good catalysts for HER, Figure 3(a,I), while with model II only V, Cr, Fe, and Co exhibit high exchange currents, Figure 3(a,II). This is because in the first case the condition Δ*G*_H_^(1)^ ∼ 0 eV is sufficient to guarantee high efficiency, while in the second one also the condition Δ*G*_H_^(2)^ ∼ 0 eV needs to be satisfied. Notice that even in Figure 3(a,II) there are metal atoms (e.g., Co) satisfying the ideal conditions for HER, i.e., Δ*G*_H_^(1)^ ∼ 0 eV and Δ*G*_H_^(2)^ ∼ 0 eV.

The other case is that of the M atoms incorporated on N-doped graphene (M@N-Gr); here, we refer to the graphitic doping configuration^87,88^ where a carbon atom of the lattice is replaced by a N atom (N-Gr) (Supporting Figures S14–S17). When we consider model I (MH intermediate only), Ti, V, Cr, Mn, Fe, Co, Ni, Cu, and Pd exhibit high currents, Figure 3(b,I), while when also HMH intermediates are considered (model II), the picture changes, with only Ti, Mn, and Fe still characterized by good performances, Figure 3(b,II) (Supporting Table S12).

The results for the remaining 33 models of SAC considered (Supporting, Figure S27) confirm this trend and show significant changes in the predicted activities when the HMH complex formation is taken into account (model II).

### Beyond Nørskov’s Model

The volcano plots discussed above represent a simple yet valuable tool to screen electrode materials for electrocatalytic processes. They are based on the combination of linear scaling relations with Sabatier’s principle and the Brønsted–Evans–Polanyi approach,^89^ which links thermodynamics to kinetics. The volcano’s apex corresponds to a material that binds the reaction intermediates neither too strongly nor too weekly at zero overpotential η (i.e., Δ*G* = 0 at η = 0). In this respect, the original model of Nørskov and co-workers,^24^ extended here to the special case of SACs, identifies the potential catalysts for which Δ*G* = 0 at η = 0. It is entirely based on a thermodynamic approach, while kinetic effects due to the presence of reaction barriers or to the applied overpotential are not included. Recently, several authors have addressed this limitation showing that there are deviations to the original model if the effect of the applied overpotential is explicitly taken into account.^25−27,90^ In particular, the most active electrocatalysts bind the reaction intermediate species with Δ*G* > 0 for η ≠ 0. This corresponds to a shift of the apex of the volcano plot on the order of 200 meV, which means that electrocatalysts that bind weakly to the surface rather than forming thermoneutral bonds can be active and should be considered as potentially good candidates.^26,27^ This shows that, for a quantitative assessment of the performance of an electrocatalyst, the effect of the overpotential, although small (≈0.2 eV), must be included.

Another aspect that is usually neglected in the modeling of materials for electrocatalysis is the solvation energy contribution, given by the interaction of the chemical intermediates with the solvent.^61,91^ In principle, ab initio molecular dynamics studies can address this problem,^92^ but they are computationally very demanding. Recently, approximated but computationally amenable recipes have been proposed to account for solvation based on microsolvation models.^93^ Furthermore, the combined effect of the applied overpotential and of the solvent can affect the morphology of the surface or induce surface reconstructions.

To assess the importance of these terms, we have introduced the effect of the solvent on the formation of MH and HMH intermediates for Co@4N-Gr, Ni@4N-G, and W@4N-Gr, using the implicit solvent approach where the water molecules are replaced by a continuum dielectric. The MH and HMH formation energies, Δ*E*^(1)^ and Δ*E*^(2)^, respectively, change by −0.03/–0.04 eV only after inclusion of the solvent (see Supporting Table S18).

Finally, HER reactions are also pH dependent.^94^ Nevertheless, in most cases the experimentally observed behavior of HER catalysts in variable pH conditions are rationalized based on DFT calculations where the pH is not explicitly taken into account.^95,96^ The pH contribution to the Gibbs free energy can be included by considering a term of the type *k*_b_*T* ln(10)pH, as reported by Nørskov and co-workers.^97,98^ Of course, one issue that remains open is the stability of the MH and HMH complexes in strong acid or basic conditions, but this is something the goes beyond the simple model to predict the HER activity.

This brief discussion shows that substantial progress has been made in recent years in the study of reactions at electrode surfaces with DFT approaches. Nørskov’s model has been used extensively in the past and will most likely continue to be used due to its simplicity. It provides a rapid screening of potentially useful catalysts at low computational cost, thus allowing the examination of large sets of new materials. What should be stressed in the present context is that things are quite different and more complex when one moves from extended metal surfaces to single-atom catalysts. As we have discussed above, the possible formation of dihydrogen and dihydride complexes results in new intermediates not considered in the original model. The formation of these intermediates can significantly alter the thermodynamics and the kinetics of the process, resulting in Δ*G* values that can differ by 1 eV or more from those obtained with the one H model. In this respect, the effects that we are discussing in this paper for SACs can dominate over other important, but quantitatively less relevant terms such as the role of the solvent and of the overpotential.

## Conclusions

We have shown that the predicted HER activity of isolated transition metal atoms supported on a solid surface, often referred to as single-atom catalysts, cannot be predicted with the standard model^24^ based on the assumption that the MH complex is the only intermediate, as for HER on metal electrodes. SACs, which can be considered analogues of coordination compounds, bind hydrogen differently and can form two-hydrogen intermediates, HMH, not present on metal surfaces. The HMH intermediate is a precursor of the H_2_ desorption step that should be taken into account in the kinetics of the process. We have extended the kinetic model proposed by Nørskov and co-workers to the case where this second intermediate forms; the resulting HER activity, as measured by the exchange current, is described by a three-dimensional volcano plot as the reaction now depends on two free energies of H adsorption instead of one. According to this new model, the condition for the best catalyst as measured by the exchange current *i*_0_ is that both Δ*G*^0^_H^(1)^_ and Δ*G*^0^_H^(2)^_ are close to zero, i.e., that the formation of H adsorbates is thermoneutral. Recent work has shown that a rigid shift of about 200 meV of the apex in a volcano curve occurs when the applied overpotential is included in the analysis.^25−27^ Nevertheless, screening studies based on the standard approach provide a first assessment of the performances of a potential new catalyst. In the case of SACs we have shown based on DFT calculations that stable dihydride and dihydrogen complexes form in many cases and that neglecting this reaction step may result in completely different and often too optimistic predictions of the catalytic activity of SACs in HER.

## Computational Methods

DFT calculations were performed using the generalized gradient approximation method with the Perdew–Burke–Ernzerhof (PBE) functional to describe electronic exchange and correlation,^99^ as implemented in the VASP code.^100,101^ A projector augmented-wave method was used to describe the core electrons. Valence electrons were described by expanding the Kohn–Sham orbitals in a plane-wave basis set, with a cutoff of 400 eV. Dispersion forces have been included according to Grimme’s parametrization.^102^ The complete computational and modeling details can be found in the Supporting Methods.

## Supporting Information Available

Details on the computational methods and of all simulated models; full set of energy data for H adsorption on various models of SACs, density of states, graphic representation of structures and geometries, vibrational frequencies of HMH complexes, and details of kinetic models; supporting figures and tables; supporting references (PDF)

## Notes

The computational data, which include the MH and HMH binding energies, the free energies of adsorption, and the optimized atomic coordinates, are available from the authors upon reasonable request.
