# Supplementary material for: Role of Dihydride and Dihydrogen Complexes in Hydrogen Evolution Reaction on Single-Atom Catalysts
Source: J Am Chem Soc. 2021 Nov 25;143(48):20431–41. doi: 10.1021/jacs.1c10470 (PMC8662730; doi:10.1021/jacs.1c10470)
Supplement: Supplementary file 1 — ja1c10470_si_001.pdf [file ja1c10470_si_001.pdf]

# Supporting information

# Role of Dihydride and Dihydrogen Complexes in Hydrogen Evolution Reaction on Single-Atom Catalysts


*Giovanni Di Liberto, Luis A. Cipriano, Gianfranco Pacchioni**

Dipartimento di Scienza dei Materiali, Università di Milano - Bicocca, via R. Cozzi 55, 20125 Milano, Italy

* Corresponding author: gianfranco.pacchioni@unimib.it


## Kinetics of HER

### A1. Volmer-Heyrovsky and Volmer-Tafel kinetics for HER on metal surfaces

The first step of the HER consists in the adsorption of a single H atom on a support, *e.g.* a metal surface, M, forming an intermediate species (MH). This step is known as Volmer step:

$$H^+ + e^- + M \rightleftarrows MH \qquad \text{Eq. (A1)}$$

Then, the evolution of $H_2$ may occur following two different paths. The first one (Heyrovsky mechanism) consists of the reduction of $H^+$, and formation of the $H_2$ molecule on the same metal site:

$$H^+ + e^- + MH \rightarrow M + H_2 \qquad \text{Eq. (A2)}$$

the second path (Tafel mechanism) consists in the combination of two MH intermediates:

$$2\,MH \rightarrow M + H_2 \qquad \text{Eq. (A3)}$$

The behavior of the exchange current can be evaluated by solving the kinetic equations associated to the Volmer-Heyrovsky and Volmer-Tafel reactions. We start from the derivation of the Volmer-Heyrovsky kinetics. One can impose the steady-state approximation for the reaction intermediate, i.e.:

$$v_1 - v_{-1} - v_2 = 0 \qquad \text{Eq. (A4)}$$

Where $v_1$ and $v_2$ are the velocities of the direct reactions in Eqs. A1 and A2. $v_{-1}$ is the velocity of the backward reaction reported in Eq. A1. A common approximation when studying kinetic models of this type consists in neglecting the backward reaction of the products formation, *i.e.* $v_{-2} = 0$.[1,2] By expanding Eq. A4 under the Butler-Volmer formalism[1] one obtains:

$$k_1[H^+][M]e^{(1-\beta)\frac{\Delta G^0{}_H}{k_bT}} - k_{-1}[MH]e^{-\beta\frac{\Delta G^0{}_H}{k_bT}} - k_2[H^+][MH]e^{-\beta\frac{\Delta G^0{}_H}{k_bT}} = 0 \qquad \text{Eq. (A5)}$$

The $\beta$ coefficient represents the symmetry of the forward and backward barriers. Usually, a common choice is $\beta = 0.5$, *i.e.* a symmetric barrier. For the forward and backward reactions, we always use $e^{(1-\beta)\frac{\Delta G^0{}_H}{k_bT}}$ and $e^{-\beta\frac{\Delta G^0{}_H}{k_bT}}$, respectively, with the exception of the products formation step (only forward reaction), for which we use $e^{-\beta\frac{\Delta G^0{}_H}{k_bT}}$.

Moreover, the "concentrations" of free metal sites $[M]$ and occupied [MH] ones are correlated by:

$$\theta_M + \theta_{MH} = 1, \qquad \text{Eq. (A6)}$$

where $\theta_M$ and $\theta_{MH}$ are the coverage fractions of free and occupied metal sites. The exchange current can be calculated as:

$$i_0 = -2ek_2[H^+]\theta_{MH}\, e^{-\beta\frac{\Delta G^0{}_H}{k_bT}} \qquad \text{Eq. (A7)}$$

If we now solve the linear system made by Eqs. A6 and A7, and we restrict to standard conditions and pH = 0, *i.e.* $p_{H_2} = 1bar$, $[H^+] = 1M$, we obtain:

$$i_0 = -2ek_2 \frac{e^{(1-\beta)\frac{\Delta G^0{}_h}{k_bT}}}{1 + e^{\frac{\Delta G^0{}_H}{k_bT}}} \qquad \text{Eq. (A8)}$$

By choosing $\beta = 0.5$ one obtains $i_0 = -2ek_2 \frac{e^{\frac{\Delta G^0{}_H}{2k_bT}}}{1+e^{\frac{\Delta G^0{}_H}{k_bT}}}$.

Similarly, we can solve the kinetic equations for the Volmer-Tafel mechanism. In this case, the steady-state approximation for the reaction intermediate MH can be written as:

$$k_1[H^+][M]e^{(1-\beta)\frac{\Delta G^0{}_H}{k_bT}} - k_{-1}[MH]e^{-\beta\frac{\Delta G^0{}_H}{k_bT}} - k_2[MH]^2e^{-\beta\frac{\Delta G^0{}_H}{k_bT}} = 0, \qquad \text{Eq. (A9)}$$

while for the coverage Eq. A6 still holds true. Solving the linear system neglecting high-order terms,[2] one obtains:

$$i_0 = -2ek_2 \left(\frac{e^{(1-\beta)\frac{\Delta G^0{}_H}{k_bT}}}{1+e^{\frac{\Delta G^0{}_H}{k_bT}}}\right)^2, \qquad \text{Eq. (A10)}$$

Once again, for $\beta = 0.5$, $i_0 = -2ek_2 \left(\frac{e^{\frac{\Delta G^0{}_H}{2k_bT}}}{1+e^{\frac{\Delta G^0{}_H}{k_bT}}}\right)^2$.

Please note that both Volmer-Heyrovsky and Volmer-Tafel mechanisms are described by a volcano curve:

$$Log(i_0) = Log(i_{max}^{VH}) + Log\left(\frac{e^{\frac{\Delta G^0{}_H}{2k_bT}}}{1+e^{\frac{\Delta G^0{}_H}{k_bT}}}\right), \quad \text{Eq. (A11)}$$

$$Log(i_0) = Log(i_{max}^{VT}) + Log\left[\left(\frac{e^{\frac{\Delta G^0{}_H}{2k_bT}}}{1+e^{\frac{\Delta G^0{}_H}{k_bT}}}\right)^2\right], \quad \text{Eq. (A12)}$$

In both cases, the maximum exchange current is obtained at $\Delta G^0{}_H = 0$, *i.e.* when the adsorption and desorption of H atoms is perfectly thermoneutral.

## A2. Kinetics of HER on single atom catalysts

Here we use the same approach described in the previous section to solve the kinetic model of HER but now accounting for the formation of two different intermediates.

$$H^+ + e^- + M \rightleftarrows MH, \quad \text{Eq. (A13)}$$

$$H^+ + e^- + MH \rightleftarrows HMH, \quad \text{Eq. (A14)}$$

$$HMH \rightarrow M + H_2. \quad \text{Eq. (A15)}$$

The first two reactions refer to the formation of the two intermediates, $MH$ and $HMH$, the third one is for $H_2$ evolution. We solve the kinetic equations by imposing the same approximations of the previous case, *i.e.* neglection of backward reaction of $H_2$ evolution, and steady-state approximation for the reaction intermediates:

$$v_1 - v_{-1} - v_2 + v_{-2} = 0, \quad \text{Eq. (A16)}$$

$$v_2 - v_{-2} - v_3 = 0. \quad \text{Eq. (A17)}$$

Please note that since two intermediates have to be considered, three equations are needed to solve the problem. The third equation accounts for the fraction of available metal sites:

$$\theta_M + \theta_{MH} + \theta_{HMH} = 1., \quad \text{Eq. (A18)}$$

If we explicit Eq. A14 and A15 we obtain:

$$k_1[H^+]\theta_M e^{(1-\beta)\frac{\Delta G^0{}_{H(1)}}{k_bT}} - k_{-1}\theta_{MH} e^{-\beta\frac{\Delta G^0{}_{H(1)}}{k_bT}} - k_2[H^+]\theta_{MH} e^{(1-\beta)\frac{\Delta G^0{}_{H(2)}}{k_bT}} + k_{-2}\theta_{HMH} e^{-\beta\frac{\Delta G^0{}_{H(2)}}{k_bT}} = 0, \quad \text{Eq. (A19)}$$

$$k_2[H^+]\theta_{MH}e^{(1-\beta)\frac{\Delta G^0{}_{H(2)}}{k_bT}} - k_{-2}\theta_{HMH}e^{-\beta\frac{\Delta G^0{}_{H(2)}}{k_bT}} - k_3[H^+]\theta_{HMH}e^{(1-\beta)\frac{\Delta G^0{}_{H(1)}+\Delta G^0{}_{H(2)}}{k_bT}} = 0, \quad \text{Eq. (A20)}$$

By solving the three variables $(\theta_M, \theta_{MH}, \theta_{HMH})$ linear system we obtain:

$$i_0 = -2ek_3 \frac{e^{(1-\beta)\frac{\left(\Delta G^0{}_{H(1)}+\Delta G^0{}_{H(2)}\right)}{k_bT}}}{1+e^{\frac{\Delta G^0{}_{H(1)}}{k_bT}}+e^{\frac{\left(\Delta G^0{}_{H(1)}+\Delta G^0{}_{H(2)}\right)}{k_bT}}+e^{\beta\frac{\Delta G^0{}_{H(1)}}{k_bT}}+e^{\beta\frac{\Delta G^0{}_{H(2)}}{2k_bT}}+e^{-\beta\frac{\Delta G^0{}_{H(1)}}{k_bT}}} \quad \text{Eq. (A21)}$$

Here, the kinetic constants have been assumed to be unitary.[1] Once again, for $\beta = 0.5$ the exchange current can be expressed as a volcano plot which is function of $\Delta G^0{}_{H(1)}$ and $\Delta G^0{}_{H(2)}$:

$$Log(i_0) = Log(i_{max}) + Log\left(\frac{e^{(1-\beta)\frac{\left(\Delta G^0{}_{H(1)}+\Delta G^0{}_{H(2)}\right)}{k_bT}}}{1+e^{\frac{\Delta G^0{}_{H(1)}}{k_bT}}+e^{\frac{\left(\Delta G^0{}_{H(1)}+\Delta G^0{}_{H(2)}\right)}{k_bT}}+e^{\beta\frac{\Delta G^0{}_{H(1)}}{k_bT}}+e^{\beta\frac{\Delta G^0{}_{H(2)}}{2k_bT}}+e^{-\beta\frac{\Delta G^0{}_{H(1)}}{k_bT}}}\right). \quad \text{Eq. (A22)}$$

The maximum exchange current is obtained when $\Delta G^0{}_{H(1)}$ and $\Delta G^0{}_{H(2)}$ are close to zero, *i.e.* when the adsorption and desorption of H atoms is thermoneutral. Please note that although the maximum current basin is when $\Delta G^0{}_{H(1)}$ and $\Delta G^0{}_{H(2)}$ are close to zero, the global maximum is not exactly at $\left(\Delta G^0{}_{H(1)}, \Delta G^0{}_{H(2)}\right) = (0,0)$, but at $(0; k_bT\ln(4)/e) \sim (0\ eV, 0.04\ eV)$. This small deviation is probably due to the approximation to consider two intermediate species under steady-state. Nevertheless, a 0.04 eV deviation is within the accuracy of DFT calculations and is not going to affect the overall picture.

## Computational details

Spin polarized DFT calculations have been performed with the VASP 6.2.0[3–5] code using the generalized gradient approximation as formulated in the Perdew–Burke–Ernzerhof (PBE) functional.[6] Dispersion contributions have been introduced using the D3 Grimme's correction.[7]

The valence electrons, C (2s,2p), N (2s, 2p), S (3s, 3p), Mo (4p, 5s, 4d), Sc (4s, 3d), Ti (4s, 3d), V (4s, 3d), Cr (4s, 3d), Mn (4s, 3d), Fe (4s, 3d), Co (4s, 3d), Ni (4s, 3d), Cu (4s, 3d), W (6s, 5d) Pd (5s, 4d), and Pt (6s, 5d), have been expanded into a set of plane waves with a kinetic cutoff of 400 eV, whereas the core electrons were treated with the projector augmented wave approach.[8,9] The truncation criteria for electronic and ionic loops were set to $10^{-6}$ eV and $10^{-3}$ eV/Å, respectively. In all cases where graphene is the support a 5×5×1 Monkhorst–Pack k-point grid was used;[10] for $MoS_2$ we reduced the k-point sampling mesh to the gamma point 1×1×1.

The hydrogen evolution reaction (HER) has been studied on first row transition metal (M) atoms either free (unsupported) or stabilized on solid surfaces: M replacing a C atom of N-graphene (M@N-Gr where the symbol @ indicates incorporation of the M atom in the graphene lattice), (4×4 supercell); a graphene model with 3N atoms that coordinate the M atom replacing a C atom (M@3N-Gr) (4×4 supercell); a M atom adsorbed on a molybdenum disulfide layered compound (M/$MoS_2$, where the symbol / indicates adsorption on the surface) (6×6 supercell); M adsorbed on N-graphene (M/N-Gr) (4x4).

For all systems considered we report the charge transfer as measured by the Quantum Theory of Atoms In Molecules (QTAIM) analysis, originally developed by Bader.[11–14] However, we will not try to use this quantity to rationalize the results as it has been shown that the Bader charges may not reflect the real charge distribution, in particular when strong covalent bonds are formed. For a discussion see e.g. refs. [15,16].

In all the studied cases, the adsorption energy of the first and second H atoms were computed as:

$$\Delta E_H = E_{MH/supp} - (E_{M/supp} + \frac{1}{2}E_{H_2})$$

$$\Delta E_H = E_{HMH/supp} - (E_{MH/supp} + \frac{1}{2}E_{H_2})$$

Where M = Sc, Ti, V, Cr, Mn, Fe, Co, Ni, Cu, Pd and Pt, and supp = support.

The total energy to adsorb two H atom on each metal atom M was computed as:

$$\Delta E_H = E_{HMH/supp} - (E_{M/supp} + E_{H_2})$$

The corresponding values are reported in square brackets in the Tables S1, S7, S10, S12, and S15.

The Gibbs free energy was computed as (see also main text):

$$\Delta G_H = \Delta E_H + \Delta E_{ZPE} - T\Delta S_H$$

## Hydrogen adsorption on M@3N-Gr

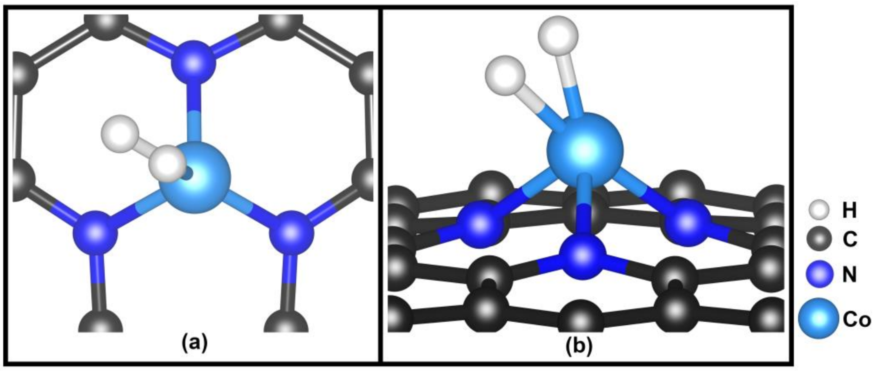


**Figure S1**. (a) top and (b) side views of HCoH@3N-Gr complex.

**Table S1**. Adsorption energy ($\Delta E_H$, in eV), Zero-Point-Energy contribution ($\Delta E_{ZPE}$, in eV), Entropy ($T\Delta S_H$, in eV), Gibbs Free Energy ($\Delta G_H$, in eV), bond distances ($d$, in Å), and bond angle (∠HMH °) for one or two H atoms adsorbed on top of M@3N-Gr (M = Sc, Ti, V, Cr, Mn, Fe, Co, Ni, Cu, Pd, and Pt). $\Delta E_H$ is the adsorption energy per H atom given with respect to ½ $H_2$; in square bracket [] is reported the energy of M + $H_2$ → HMH.

| | $\Delta E_H$ | $\Delta E_{ZPE}$ | $T\Delta S_H$ | $\Delta G_H$ | $d_{N-M}$ | $d_{M-H}$ | $d_{H-H}$ | ∠H-M-H |
|---|---|---|---|---|---|---|---|---|
| $H_2$ | --- | --- | --- | --- | --- | --- | 0.75 | ---- |

| | | | | | | | | |
|---|---|---|---|---|---|---|---|---|
| ScH@3N-Gr | -0.79 | 0.009 | -0.19 | -0.59 | 2.06 | 1.88 | --- | --- |
| HScH@3N-Gr | 0.61 [-0.18] | 0.027 | -0.38 | 0.82 [0.23] | 2.13-2.23 | 1.86 | 3.06 | 111 |
| TiH@3N-Gr | -0.89 | 0.023 | -0.19 | -0.68 | 1.96 | 1.78 | --- | --- |
| HTiH@3N-Gr | 0.25 [-0.64] | 0.066 | -0.38 | 0.49 [-0.19] | 1.96-2.01 | 1.73-1.75 | 2.38 | 86 |
| VH@3N-Gr | -1.42 | 0.028 | -0.19 | -1.20 | 1.90 | 1.71 | --- | --- |
| HVH@3N-Gr | 0.55 [-0.87] | 0.083 | -0.38 | 0.79 [-0.41] | 1.89-1.94 | 1.67 | 1.70 | 61 |
| CrH@3N-Gr | -0.54 | 0.017 | -0.19 | -0.33 | 1.91 | 1.70 | --- | --- |
| HCrH@3N-Gr | 1.20 [0.66] | 0.060 | -0.38 | 1.43 [1.10] | 1.93-2.04 | 1.65 | 2.28 | 88 |
| MnH@3N-Gr | -0.53 | 0.024 | -0.19 | -0.32 | 1.79 | 1.60 | --- | --- |
| HMnH@3N-Gr | 0.09 [-0.64] | 0.147 | -0.38 | 0.21 [-0.11] | ~1.83 | 1.66-1.70 | 0.88 | 30 |
| FeH@3N-Gr | -0.50 | 0.022 | -0.19 | -0.29 | 1.97 | 1.60 | --- | --- |
| HFeH@3N-Gr | -0.30 [-0.80] | 0.106 | -0.38 | -0.02 [-0.31] | 1.90-1.93 | 1.65 | 0.91 | 32 |
| CoH@3N-Gr | -0.34 | 0.027 | -0.19 | -0.12 | 1.90 | 1.56 | --- | --- |
| HCoH@3N-Gr | -0.44 [-0.78] | 0.128 | -0.38 | -0.15 [-0.27] | 1.88-1.92 | ~1.60 | 0.90 | 32 |
| NiH@3N-Gr | -0.44 | 0.050 | -0.19 | -0.20 | 1.97 | 1.55 | --- | --- |
| HNiH@3N-Gr | -0.64 [-1.08] | 0.129 | -0.38 | -0.37 [-0.57] | 1.94 | 1.53 | 1.01 | 38 |
| CuH@3N-Gr | -0.05 | 0.040 | -0.19 | 0.18 | 1.93-2.04 | 1.50 | --- | --- |
| HCuH@3N-Gr | -1.04 [-1.09] | 0.145 | -0.38 | -0.75 [-0.57] | 1.97-2.04 | 1.57 | 0.88 | 33 |
| PdH@3N-Gr | -0.50 | 0.083 | -0.19 | -0.23 | 2.06-2.35 | 1.57 | --- | --- |
| HPdH@3N-Gr | -0.69 [-1.19] | 0.147 | -0.38 | -0.43 [-0.66] | 2.14 | 1.56 | 1.82 | 71 |
| PtH@3N-Gr | -1.45 | 0.084 | -0.19 | -1.18 | 2.14-2.15 | 1.59 | --- | --- |
| HPtH@3N-Gr | -1.41 [-2.86] | 0.194 | -0.38 | -1.11 [-2.29] | 2.14-2.15 | 1.57 | 2.00 | 79 |

## Hydrogen adsorption on Co/$MoS_2$

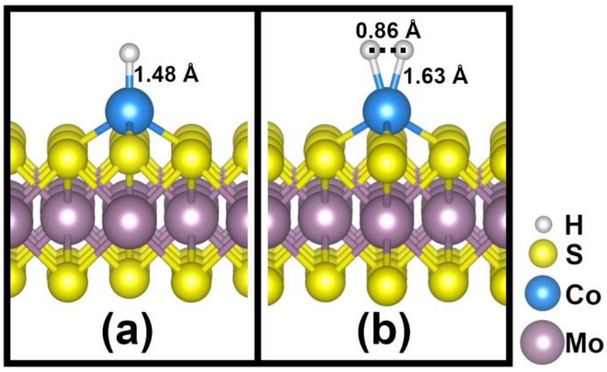


**Figure S2 –** Structure of CoH/$MoS_2$ and HCoH/$MoS_2$ complexes. The H-H distance in HCoH/$MoS_2$ is 0.86 Å

**Table S2:** Calculated Gibbs free energy changes for the adsorption of the first hydrogen atom ($\Delta G^{(1)}$), the second hydrogen atom ($\Delta G^{(2)}$), and two hydrogen atoms ($\Delta G^{(3)} = \Delta G^{(1)} + \Delta G^{(2)}$). Calculated overpotential by considering the formation of one intermediate, MH ($\eta^{MH}$), and both MH and HMH intermediates ($\eta^{HMH}$). The measured overpotential is reported for comparison ($\eta^{exp}$). Experimental data are taken from Ref. [17].

| system | | $\Delta G^{(1)}$/ meV | $\Delta G^{(2)}$/meV | $\Delta G^{(3)}$/meV | | $\eta^{MH}$/mV | $\eta^{HMH}$/mV | $\eta^{exp}$/mV |
|---|---|---|---|---|---|---|---|---|
| Co/$MoS_2$ | Top Mo | 340 | -665 | -324 | | 340 | 162 | 83 |
| Co/$MoS_2$ | hollow | 269 | -387 | -118 | | 269 | 59 | 83 |

## Hydrogen adsorption on Ni@D-Gr

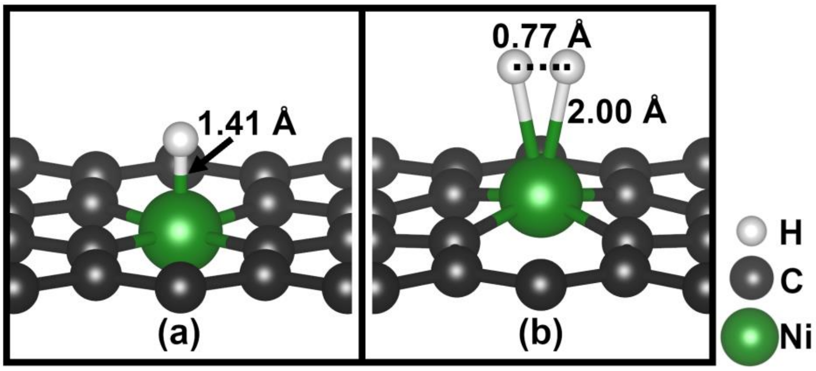


**Figure S3 –** Structure of NiH@D-Gr and HNiH@D-Gr complexes. The H-H distance in HNiH@D-Gr is 0.77 Å

**Table S3:** Calculated Gibbs free energy changes for the adsorption of the first hydrogen atom ($\Delta G^{(1)}$), the second hydrogen atom ($\Delta G^{(2)}$), and two hydrogen atoms ($\Delta G^{(3)} = \Delta G^{(1)} + \Delta G^{(2)}$). Calculated overpotential by considering the formation of one intermediate, MH ($\eta^{MH}$), and both MH and HMH intermediates ($\eta^{HMH}$). The measured overpotential is reported for comparison ($\eta^{exp}$). Experimental data are taken from Ref. [18].

| System | $\Delta G^{(1)}$/ meV | $\Delta G^{(2)}$/meV | $\Delta G^{(3)}$/meV | | $\eta^{MH}$/mV | $\eta^{HMH}$/mV | $\eta^{exp}$/mV |
|---|---|---|---|---|---|---|---|
| Ni@D-Gr | 464 | -68 | 394 | | 464 | 197 | 70 |

## Hydrogen adsorption on M@4N-Gr (M = Co, W, Ni)

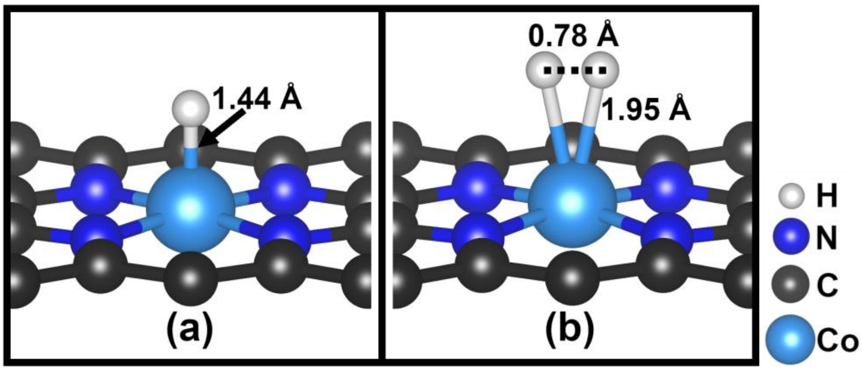


**Figure S4 –** Structure of CoH@4N-Gr and HCoH@4N-Gr complexes. The H-H distance in HCoH@4N-Gr is 0.78 Å

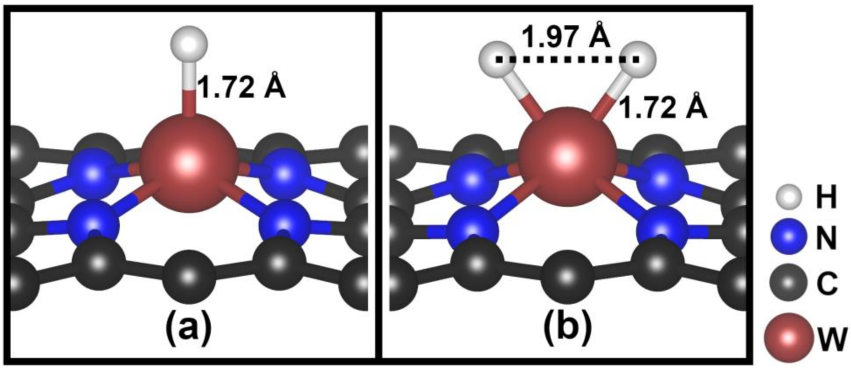


**Figure S5** – Structure of WH@4N-Gr and HWH@4N-Gr complexes. The H-H distance in HWH@4N-Gr is 1.97 Å

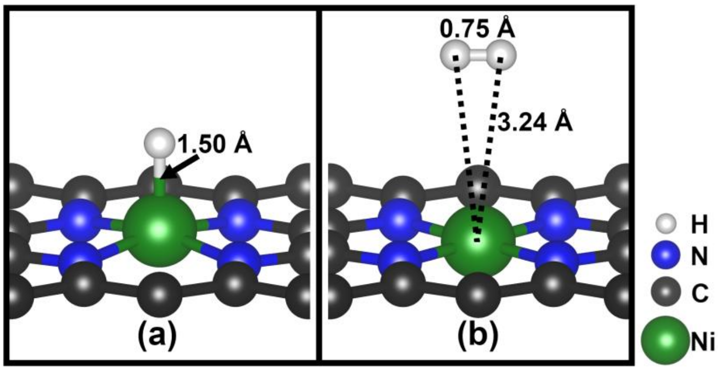


**Figure S6** – Structure of NiH@4N-Gr and HNiH@4N-Gr complexes. The H-H distance in HNiH@4N-Gr is 0.75 Å

**Table S4:** Calculated Gibbs free energy changes for the adsorption of the first hydrogen atom ($\Delta G^{(1)}$), the second hydrogen atom ($\Delta G^{(2)}$), and two hydrogen atoms ($\Delta G^{(3)} = \Delta G^{(1)} + \Delta G^{(2)}$). Calculated overpotential by considering the formation of one intermediate, MH ($\eta^{MH}$), and both MH and HMH intermediates ($\eta^{HMH}$). The measured overpotential is reported for comparison ($\eta^{exp}$). Experimental data are taken from Ref. [19].

| Metal | $\Delta G^{(1)}$/ meV | $\Delta G^{(2)}$/meV | $\Delta G^{(3)}$/meV | | $\eta^{MH}$/mV | $\eta^{HMH}$/mV | $\eta^{exp}$/mV |
|---|---|---|---|---|---|---|---|
| Co@4N-Gr | 162 | 352 | 514 | | 162 | 257 | 230 |
| W@4N-Gr | -882 | 5 | -877 | | 882 | 439 | 530 |
| Ni@4N-Gr | 1614 | / | / | | 1614 | / | 590 |

## Effect of exchange-correlation functional on the HER of M@4N-Gr (M = Co, W, Ni)

In Table S5 are reported the adsorption energies of the first and second hydrogen adsorbed on a metal (Co, Ni, W) in a carbon divacancy of N-Gr. The TM atom is coordinated to four nitrogen atoms(M@4N-Gr), Figure S7. The geometries and adsorption energies were calculated using three different functionals, PBE, PBESol, and revPBE as:

$$\Delta E^{(1)} = E_{MH@4N-Gr} - (E_{M@4N-Gr} + 1/2E_{H_2})$$
$$\Delta E^{(2)} = E_{HMH@4N-Gr} - (E_{MH@4N-Gr} + 1/2E_{H_2})$$
$$\Delta E^{(3)} = E_{HMH@4N-Gr} - (E_{M@4N-Gr} + E_{H_2}) = \Delta E^{(1)} + \Delta E^{(2)}$$

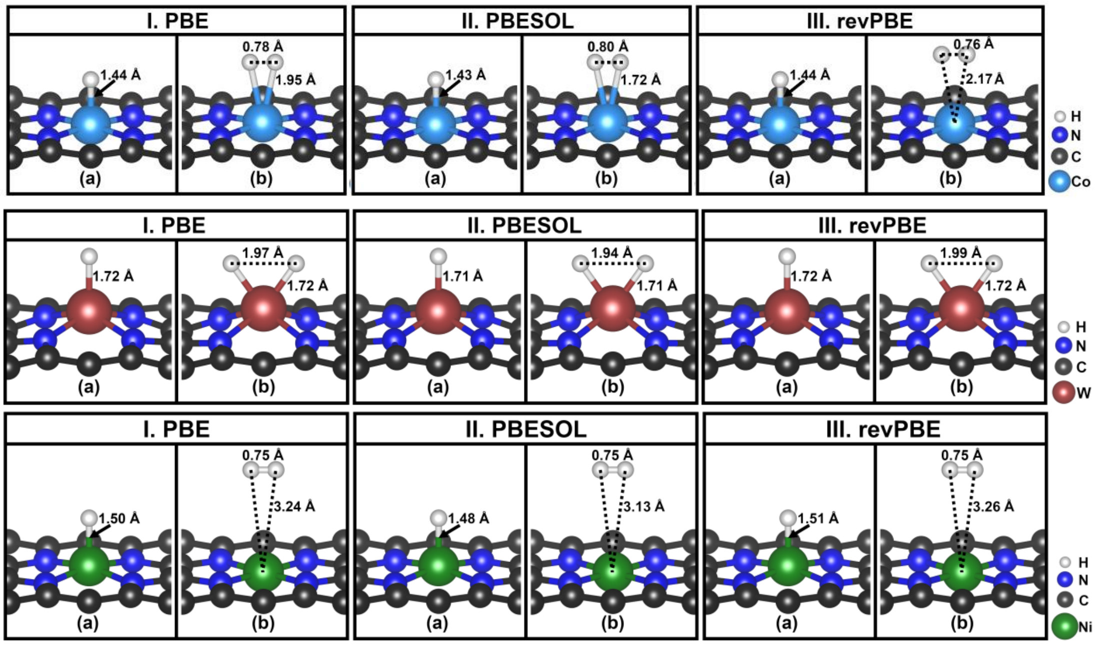


**Figure S7**. Structures of nine MH and HMH complexes of M@4N-Gr computed with PBE, PBESOL, and revPBE functionals.

**Table S5**. Adsorption energies of the first and second hydrogen on M@4N-Gr support using different functionals. Values in meV.

| Metal | functional | $\Delta E^{(1)}$ / meV | $\Delta E^{(2)}$ / meV | $\Delta E^{(3)}$ / meV |
|---|---|---|---|---|
| Co@4N-Gr | PBE | -108 | 123 | 15 |
| | PBESOL | -283 | 163 | -120 |
| | revPBE | -23 | 71 | 48 |
| | | | | |
| W@4N-Gr | PBE | -1122 | -245 | -1367 |
| | PBESOL | -1205 | -304 | -1509 |
| | revPBE | -1010 | -230 | -1240 |
| | | | | |
| Ni@4N-Gr | PBE | 1414 | / | / |
| | PBESOL | 1320 | / | / |
| | revPBE | 1452 | / | / |

In Table S6 is reported a comparison between the experimental over-potentials and the computed Gibbs free energies computed using $\Delta E_{ZPE}$ and $\Delta TS_H$ as in the original work of Nørskov and collaborators. The Gibbs free energies were calculated as:

$$\Delta G^{(1)} = \Delta E^{(1)} + 0.24$$
$$\Delta G^{(2)} = \Delta E^{(2)} + 0.24$$
$$\Delta G^{(3)} = \Delta E^{(3)} + 0.48 = \Delta G^{(1)} + \Delta G^{(2)}$$

**Table S6**. Gibbs free energies of the first and second hydrogen adsorbed on M@4N-Gr using different functionals. Values in meV.

| Metal | functional | $\Delta G^{(1)}$ / meV | $\Delta G^{(2)}$ / meV | $\Delta G^{(3)}$ / meV | | $\eta^{MH}$ / mV | $\eta^{HMH}$ / mV | $H^{exp}$ / mV |
|---|---|---|---|---|---|---|---|---|
| Co@4N-Gr | PBE | 132 | 363 | 495 | | 132 | 248 | 230 |
| | PBESOL | -43 | 403 | 360 | | 43 | 180 | |
| | revPBE | 217 | 311 | 528 | | 217 | 264 | |
| | | | | | | | | |
| W@4N-Gr | PBE | -882 | -5 | -887 | | 882 | 444 | 530 |
| | PBESOL | -965 | -64 | -1029 | | 965 | 515 | |
| | revPBE | -770 | 10 | -760 | | 770 | 380 | |
| | | | | | | | | |
| Ni@4N-Gr | PBE | 1654 | / | / | | 1654 | / | 590 |
| | PBESOL | 1560 | / | / | | 1560 | | |
| | revPBE | 1692 | / | / | | 1692 | | |

## Hydrogen adsorption on free M atoms

**Table S7.** Adsorption energy ($\Delta E_{H}$, in eV), Zero-Point-Energy contribution ($\Delta E_{ZPE}$, in eV), Entropy ($T\Delta S_{H}$, in eV), Gibbs Free Energy ($\Delta G_{H}$, in eV), bond distances (*d*, in Å), and bond angle (∠HMH °) for one or two H atoms adsorbed on M (M = Sc, Ti, V, Cr, Mn, Fe, Co, Ni, Cu, Pd, and Pt). $\Delta E_{H}$ is the adsorption energy per H atom given with respect to ½ $H_2$; in square bracket [] is reported the energy of M + $H_2$ → HMH.

| | $\Delta E_{H}$ | $\Delta E_{ZPE}$ | $T\Delta S_{H}$ | $\Delta G_{H}$ | $d_{M-H}$ | $d_{H-H}$ | ∠HMH |
|---|---|---|---|---|---|---|---|
| $H_2$ | --- | --- | --- | --- | --- | 0.75 | --- |
| ScH | -0.19 | -0.027 | -0.19 | -0.03 | 1.77 | --- | --- |
| HScH | -0.78 [-0.97] | -0.048 | -0.38 | -0.61 [-0.64] | 1.81 | 3.05 | 115 |
| TiH | -0.28 | -0.038 | -0.19 | -0.13 | 1.75 | --- | --- |
| HTiH | -0.69 [-0.97] | -0.033 | -0.38 | -0.50 [-0.63] | 1.75 | 3 | 118 |
| VH | -0.24 | -0.035 | -0.19 | -0.09 | 1.68 | --- | --- |
| HVH | -0.22 [-0.46] | -0.028 | -0.38 | -0.02 [-0.11] | 1.68 | 2.85 | 116 |
| CrH | -0.05 | -0.032 | -0.19 | 0.11 | 1.64 | --- | --- |
| HCrH | 0.04 [-0.01] | -0.022 | -0.38 | 0.24 [0.35] | 1.63 | 2.69 | 111 |
| MnH | 0.22 | -0.027 | -0.19 | 0.38 | 1.58 | --- | --- |
| HMnH | -0.73 [-0.51] | -0.039 | -0.38 | -0.55 [-0.17] | 1.66 | 3.25 | 156 |
| FeH | 0.06 | -0.027 | -0.19 | 0.22 | 1.56 | --- | --- |
| HFeH | -0.08 [-0.02] | 0.005 | -0.38 | 0.14 [0.37] | 1.51 | 2.29 | 98 |
| CoH | -0.23 | -0.014 | -0.19 | -0.05 | 1.48 | --- | --- |
| HCoH | -0.36 [-0.59] | 0.019 | -0.38 | -0.14 [ -0.19] | 1.46 | 2.13 | 94 |
| NiH | -0.96 | -0.013 | -0.19 | -0.78 | 1.46 | --- | --- |
| HNiH | -0.38 [-1.34] | 0.030 | -0.38 | -0.15 [-0.93] | 1.42 | 1.8 | 79 |
| CuH | -0.64 | -0.013 | -0.19 | -0.46 | 1.46 | --- | --- |
| HCuH | 0.71 [0.07] | -0.013 | -0.38 | 0.90 [0.44] | 1.51 | 2.68 | 125 |
| PdH | -0.34 | -0.010 | -0.19 | -0.16 | 1.54 | --- | --- |

| | | | | | | | |
|---|---|---|---|---|---|---|---|
| HPdH | -0.73 [-1.07] | 0.021 | -0.38 | -0.51 [-0.67] | 1.52 | 1.75 | 70 |
| PtH | -1.37 | 0.011 | -0.19 | -1.02 | 1.53 | --- | --- |
| HPtH | -1.39 [-2.76] | 0.077 | -0.38 | -1.13 [-2.30] | 1.52 | 2.06 | 86 |

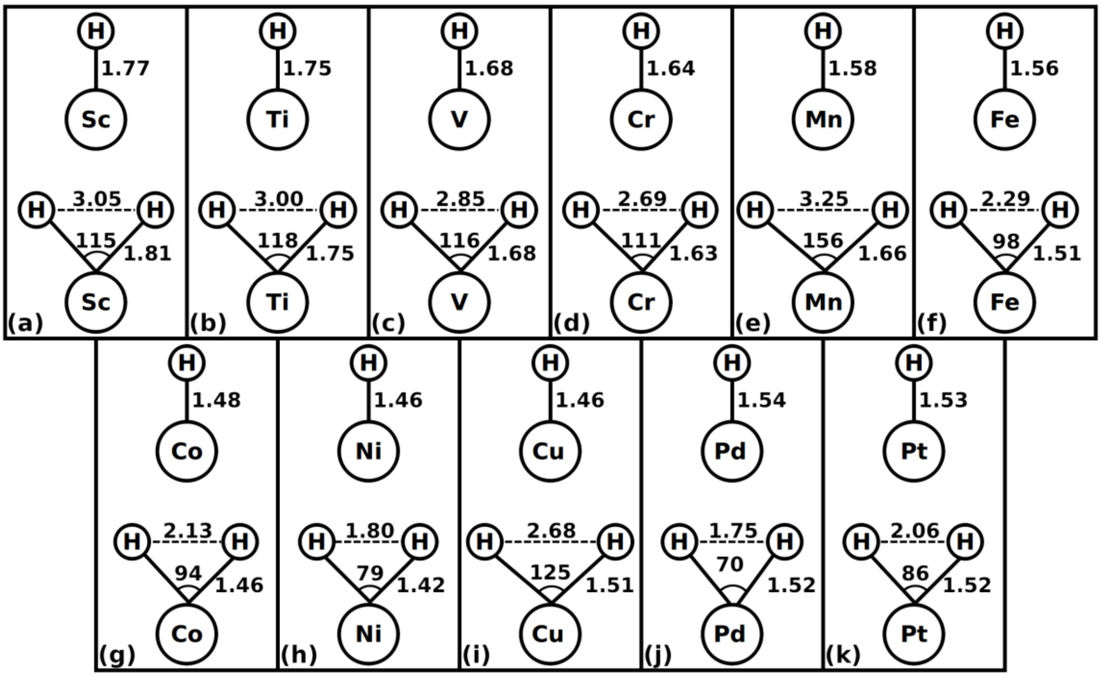


**Figure S8.** Schematic view of the MH and HMH intermediates for free M atoms. Bond lengths are reported in Å, bond angles in degrees (º). (a) ScH, HScH; (b) TiH, HTiH; (c) VH, HVH; (d) CrH, HCrH; (e) MnH, HMnH; (f) FeH, HFeH; (g) CoH, HCoH; (h) NiH, HNiH; (i) CuH, HCuH; (j) PdH, HPdH; and (k) PtH, HPtH.

## Transition metal atoms on N-doped graphene (M/N-Gr)

For each transition metal atom, we have considered at least 5 adsorption sites on the N-Gr support: on top of N, $C_{ortho}$, $C_{meta}$, $C_{para}$ and on-top of a C atom far from the N-dopant ($C_{25}$), see Figure S9a. After geometry optimization we found that almost all the metal atoms considered move to the hollow sites, which are energetically more favorable. For Sc, Ti and V the most stable site is the Hollow 2 (H2), for Cr, Mn, Fe, Co, and Ni the Hollow 1 (H1), while Pd and Pt prefer a bridge site (B); for the Cu atom the $C_{ortho}$ site is the most stable, see Figure S9b. Notice that after adsorbing the metal atoms the N-Gr support remains flat.

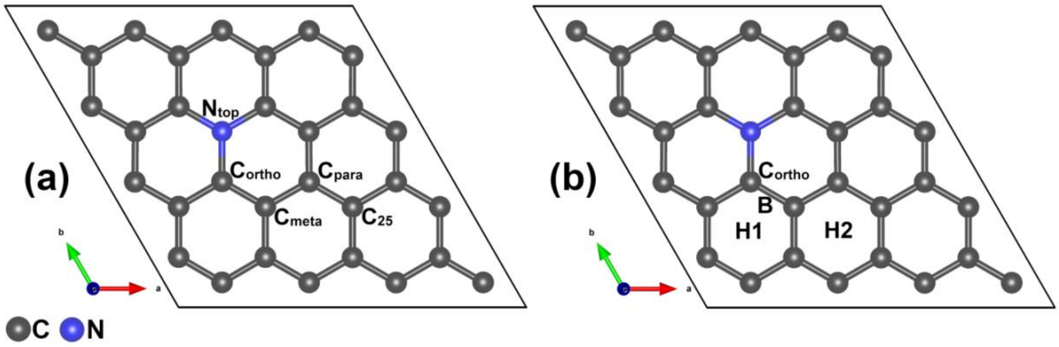


**Figure S9**. (a) definition of the adsorption sites considered as initial sites, (b) the most stable sites to adsorb the transition metal atoms.

In Table S8 we report the binding energy (BE) (in eV), the distance between C and each M atom, the charge transfer (CT) from M to N-Gr (in |e|) as measured by the QTAIM), and the electronic configuration of the M atom adsorbed on N-Gr. The *s* and *d* orbital occupancy were obtained from the integration of the Atomic Projected Density of States (PDOS) curves, see Figure S10. The binding energy was computed as:

$$\mathrm{BE} = \mathrm{M/N\text{–}Gr} - (\mathrm{N\text{–}Gr} + \mathrm{M})$$

**Table S8**. Binding energy (in eV), bond distance (in Å), charge transfer (CT) from M to N-Gr (in |e|), and electronic configuration of the M atom (PBE/D3 results).

| M | Site | BE | $d_{\text{C-M}}$ | CT | 4s | 3d |
|---|---|---|---|---|---|---|
| Sc/N-Gr | H2 | -1.73 | 2.39-2.42 | 0.82 | 0.92 | 1.54 |
| Ti/N-Gr | | -1.94 | 2.30-2.32 | 0.81 | 0.84 | 2.75 |
| V/N-Gr | | -1.31 | 2.28-2.35 | 0.68 | 0.73 | 3.81 |
| Cr/N-Gr | H1 | -0.52 | 2.59 | 0.27 | 0.82 | 4.69 |
| Mn/N-Gr | | -0.31 | 2.24-2.46 | 0.48 | 0.91 | 5.16 |
| Fe/N-Gr | | -1.03 | 2.08-2.17 | 0.58 | 0.77 | 6.87 |
| Co/N-Gr | | 0.20 | 2.03-2.13 | 0.49 | 0.74 | 8.14 |
| Ni/N-Gr | | -1.52 | 1.97-2.07 | 0.37 | 0.70 | 9.10 |
| Cu/N-Gr | $C_{ortho}$ | -0.45 | 2.02 | 0.07 | 1.33 | 9.65 |
| Pd/N-Gr | B | -1.39 | 2.15 | -0.13 | 0.64 | 9.27 |
| Pt/N-Gr | | -2.22 | 2.05-2.12 | 0.09 | 1.20 | 9.00 |

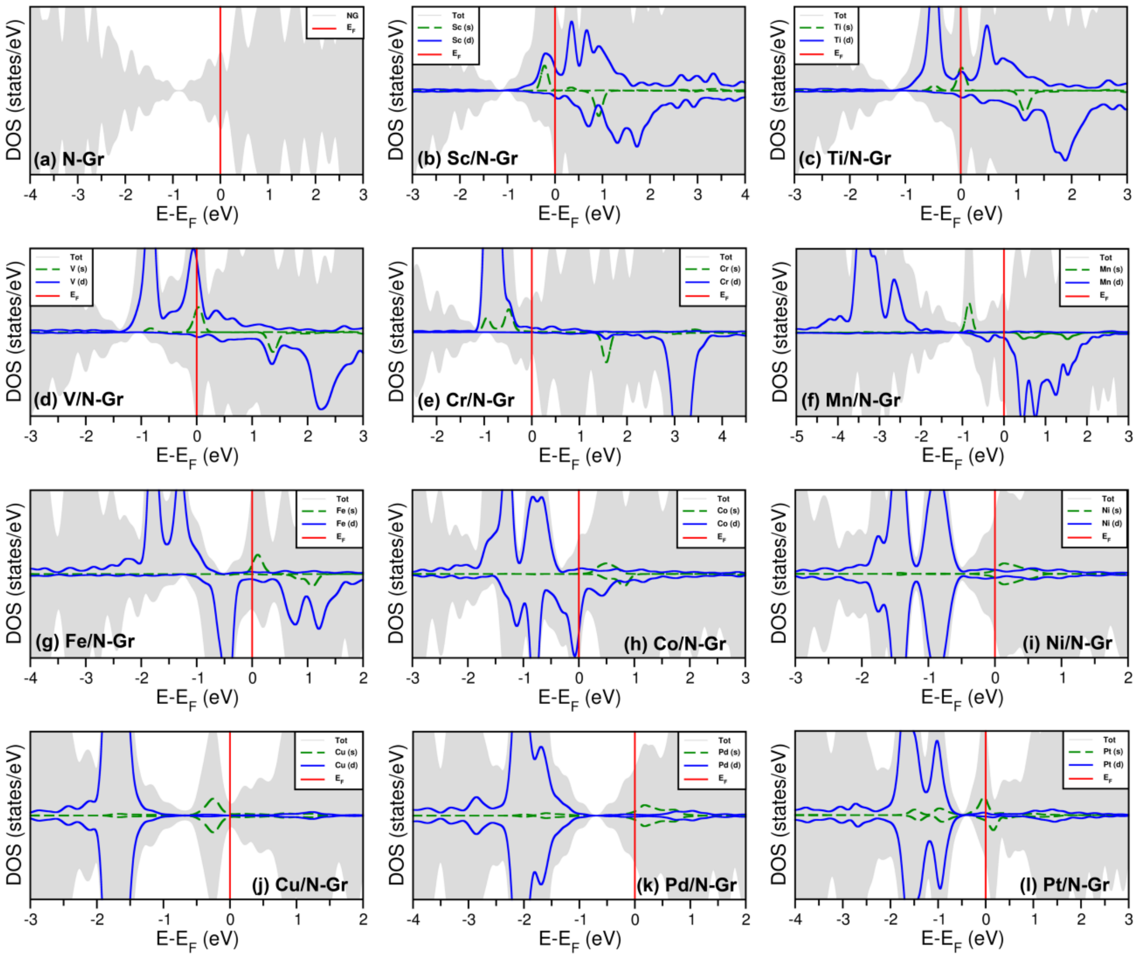

**Figure S10**. Partial density of states of the M atoms adsorbed on N-Gr. (a) N-Gr, (b) Sc/N-Gr, (c) Ti/N-Gr, (d) V/N-Gr, (e) Cr/N-Gr, (f) Mn/N-Gr, (g) Fe/N-Gr, (h) Co/N-Gr, (i) Ni/N-Gr, (j) Cu/N-Gr, (k) Pd/N-Gr, and (l) Pt/N-Gr. The gray shadow line represents the total density of states, the green dotted line the states due to the 4s orbital, the blue line the states due to the 3d orbitals, and the red line denoted the Fermi level.

## H adsorption on M/N-Gr

Before considering the adsorption of H on the M atoms adsorbed on a support, we have considered H adsorption on the pristine support. When H is adsorbed on top of C, it forms a C-H bond of about ~1.12 Å, and the C atom moves up from the N-Gr plane by ~0.47-0.58 Å. When H is adsorbed on top of a N atom, it forms a N-H bond of 1.05 Å and the N atom protrudes from the N-Gr plane 0.48 Å. In Table S9 we report the adsorption energy (in eV), bond distance (in Å), and dipole moment (in a.u) of H adsorbed on N-Gr.

**Table S9.** Binding energy ($\Delta E_H$ in eV), bond distance (*d* in Å), and dipole moment ($\mu$ in a.u) for a H atom adsorbed on N-Gr.

| site | $\Delta E_H$ | $d_{X-H}$ (X=N,C) | $\mu$ |
|---|---|---|---|
| N | 1.62 | 1.05 | 0.00 |
| $C_{ortho}$ | 0.41 | 1.12 | 0.28 |
| $C_{meta}$ | 1.01 | 1.13 | 0.29 |

| | | | |
|---|---|---|---|
| $C_{para}$ | 0.66 | 1.12 | 0.28 |
| $C_{25}$ | 1.12 | 1.13 | 0.32 |

We studied the adsorption of one and two H atoms on the most stable models of SACs for M/N-Gr. After we adsorbed the first H atom, we considered various adsorption positions and we found that the most stable site one is always on-top of the metal. Depending on the metal atom M, when H is adsorbed on-top of N, the N atom moves up by ~0.45 Å to form a bond with H (N-H bond length ~ 1.00 Å). Since the most stable adsorption site for H is on-top of M, we adsorbed the second H in this site. In almost all cases the N-Gr support remains flat, except for Cu/N-Gr, where the $C_{ortho}$ atom moves up by ~0.35 Å from the N-Gr plane. When we adsorbed the second H on Pd/N-Gr and Pt/N-Gr, the Pd and Pt atoms moved spontaneously from the bridge to the $C_{meta}$ and $C_{ortho}$ adsorption site, respectively.

**Table S10.** Adsorption energy ($\Delta E_H$, in eV), Zero-Point-Energy contribution ($\Delta E_{ZPE}$, in eV), Entropy ($T\Delta S_H$, in eV), Gibbs Free Energy ($\Delta G_H$, in eV), bond distances ($d$, in Å), and bond angle (∠HMH °) for one or two H atoms adsorbed on top of M/N-Gr (M = Sc, Ti, V, Cr, Mn, Fe, Co, Ni, Cu, Pd, and Pt). $\Delta E_H$ is the adsorption energy per H atom given with respect to ½ $H_2$; in square bracket [] is reported the energy of M + $H_2$ → HMH.

| Site | $\Delta E_H$ | $\Delta E_{ZPE}$ | $T\Delta S_H$ | $\Delta G_H$ | $d_{C\text{-}M}$ | $d_{M\text{-}H}$ | $d_{H\text{-}H}$ | ∠H-M-H |
|---|---|---|---|---|---|---|---|---|
| $H_2$ | --- | --- | --- | --- | --- | --- | 0.75 | --- |
| ScH/N-Gr | -0.85 | 0.006 | -0.19 | -0.65 | 2.33-2.41 | 1.86 | --- | --- |
| HScH/N-Gr | -0.09 [-0.94] | 0.040 | -0.38 | 0.13 [-0.52] | 2.46-2.51 | 1.83 | 3.10 | 116 |
| TiH/N-G | -0.64 | 0.008 | -0.19 | -0.44 | 2.20-2.25 | 1.76 | --- | --- |
| HTiH/N-Gr | -0.10 [-0.74] | 0.047 | -0.38 | 0.13 [-0.31] | 2.28-2.32 | ~1.75 | 2.89 | 112 |
| VH/N-Gr | -0.70 | 0.019 | -0.19 | -0.49 | 2.23-2.26 | 1.71 | --- | --- |
| HVH/N-Gr | -0.10 [-0.80] | 0.053 | -0.38 | 0.12 [-0.37] | 2.32-2.36 | ~1.68 | 2.66 | 105 |
| CrH/N-Gr | -0.38 | 0.014 | -0.19 | -0.18 | 2.30 | 1.67 | --- | --- |
| HCrH/N-Gr | -0.05 [-0.43] | 0.034 | -0.38 | 0.16 [-0.02] | 2.36-2.47 | 1.63 | 2.40 | 95 |
| MnH/N-Gr | -0.68 | 0.013 | -0.19 | -0.48 | 2.25-2.42 | 1.62 | --- | --- |
| HMnH/N-Gr | 0.03 [-0.65] | 0.111 | -0.38 | 0.32 [-0.16] | 2.21-2.32 | 1.58 | 1.96 | 77 |
| Fe/NGr | -0.52 | -0.015 | -0.19 | -0.35 | 2.16-2.21 | 1.59 | --- | --- |
| HFeH/NGr | -0.26 [-0.78] | 0.030 | -0.38 | -0.02 [-0.37] | 2.10-2.20 | 1.53 | 1.78 | 72 |
| CoH/N-Gr | -0.68 | 0.066 | -0.19 | -0.42 | 2.15-2.25 | 1.55 | --- | --- |
| HCoH/N-Gr | -0.72 [-1.40] | 0.109 | -0.38 | -0.49 [-0.91] | 2.05-2.14 | 1.55 | 0.96 | 36 |
| NiH/N-Gr | -0.57 | 0.045 | -0.19 | -0.34 | 1.99-2.15 | 1.53 | --- | --- |
| HNiH/N-Gr | -0.90 [-1.47] | 0.133 | -0.38 | -0.62 [-0.96] | 2.14-2.20 | 1.54 | 0.91 | 35 |
| CuH/N-Gr | -0.74 | 0.052 | -0.19 | -0.50 | 2.10 | 1.49 | --- | --- |
| HCuH/NGr | 0.90 [0.16] | 0.106 | -0.38 | 1.15 [0.65] | 2.31 | 1.53 | 3.06 | 171 |
| PdH/N-Gr | -0.20 | 0.039 | -0.19 | 0.03 | 2.07-2.26 | 1.60 | --- | --- |
| HPdH/N-Gr | -0.78 [-0.98] | 0.145 | -0.38 | -0.58 [-0.55] | 2.15 | 1.70 | 0.88 | 30 |
| PtH/N-Gr | -1.08 | 0.065 | -0.19 | -0.83 | 2.04-2.28 | 1.59 | --- | --- |
| HPtH/N-Gr | -0.50 [-1.58] | 0.149 | -0.38 | -0.22 [-1.05] | 2.21 | 1.55 | 1.84 | 73 |

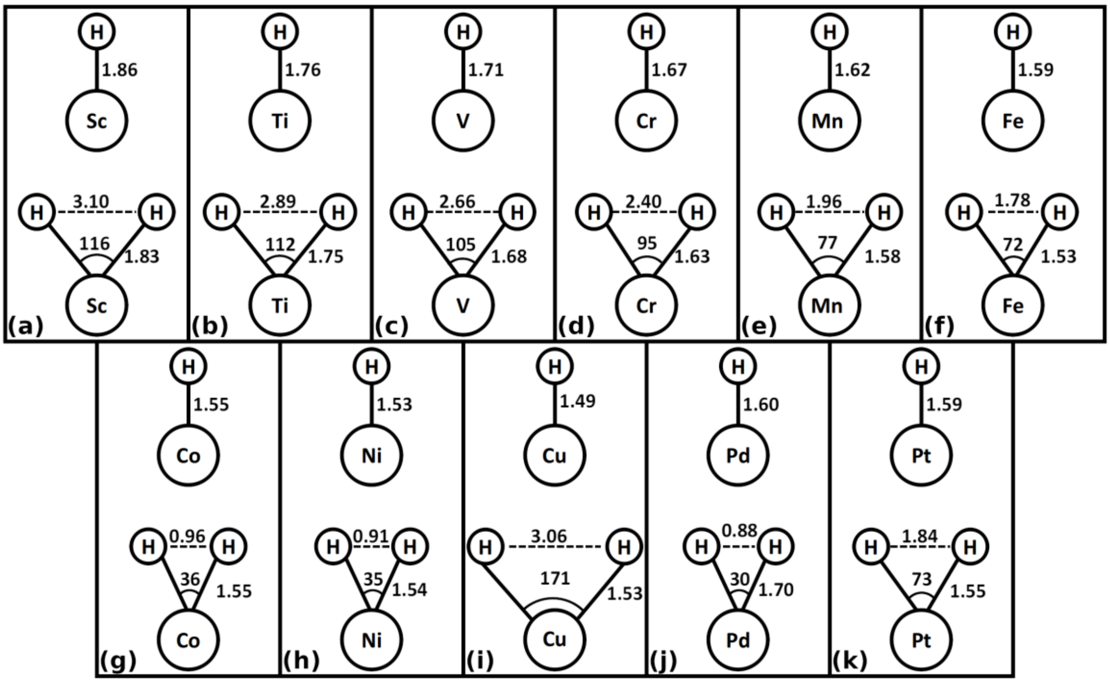


**Figure S11**. Schematic view of the MH/N-Gr and HMH/N-Gr intermediates. Bond lengths are reported in Å, bond angles in degrees (º). (a) HSc/N-Gr, HScH/N-Gr; (b) TiH/N-Gr, HTiH/N-Gr; (c) VH/N-Gr, HVH/N-Gr; (d) CrH/N-Gr, HCrH/N-Gr; (e) MnH/N-Gr, HMnH/N-Gr; (f) FeH/N-Gr, HFeH/N-Gr; (g) CoH/N-Gr, HCoH/N-Gr; (h) NiH/N-Gr, HNiH/N-Gr; (i) CuH/N-Gr, HCuH/N-Gr; (j) PdH/N-Gr, HPdH/N-Gr; and (k) PtH/N-Gr, HPtH/N-Gr.

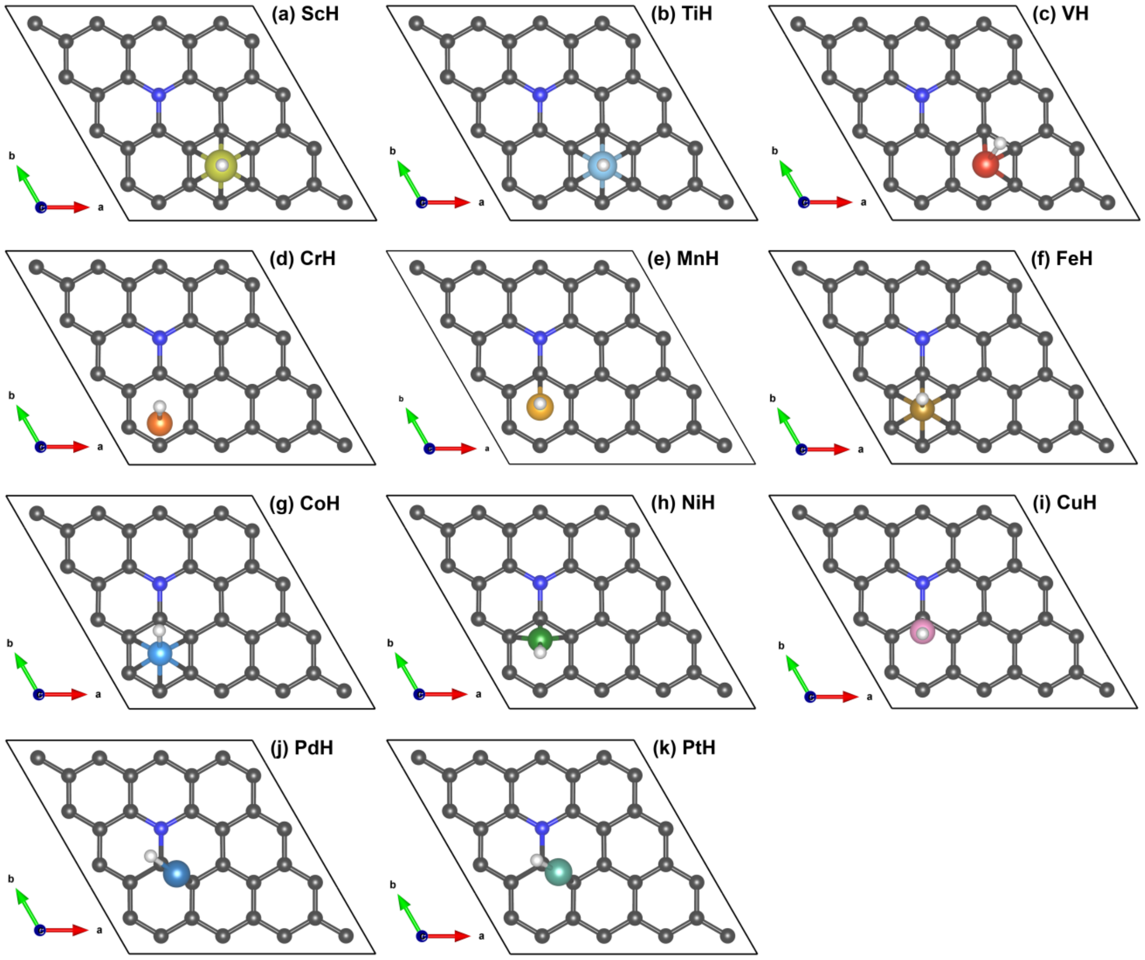


**Figure S12**. Top views of the first H adsorption on M/N-Gr. (a) ScH/N-Gr, (b) TiH/N-Gr, (c) VH/N-Gr, (d) CrH/N-Gr, (e) MnH/N-Gr, (f) FeH/N-Gr, (g) CoH/N-Gr, (h) NiH/N-Gr, (i) CuH/N-Gr, (j) PdH/N-Gr, and (k) PtH/N-Gr.

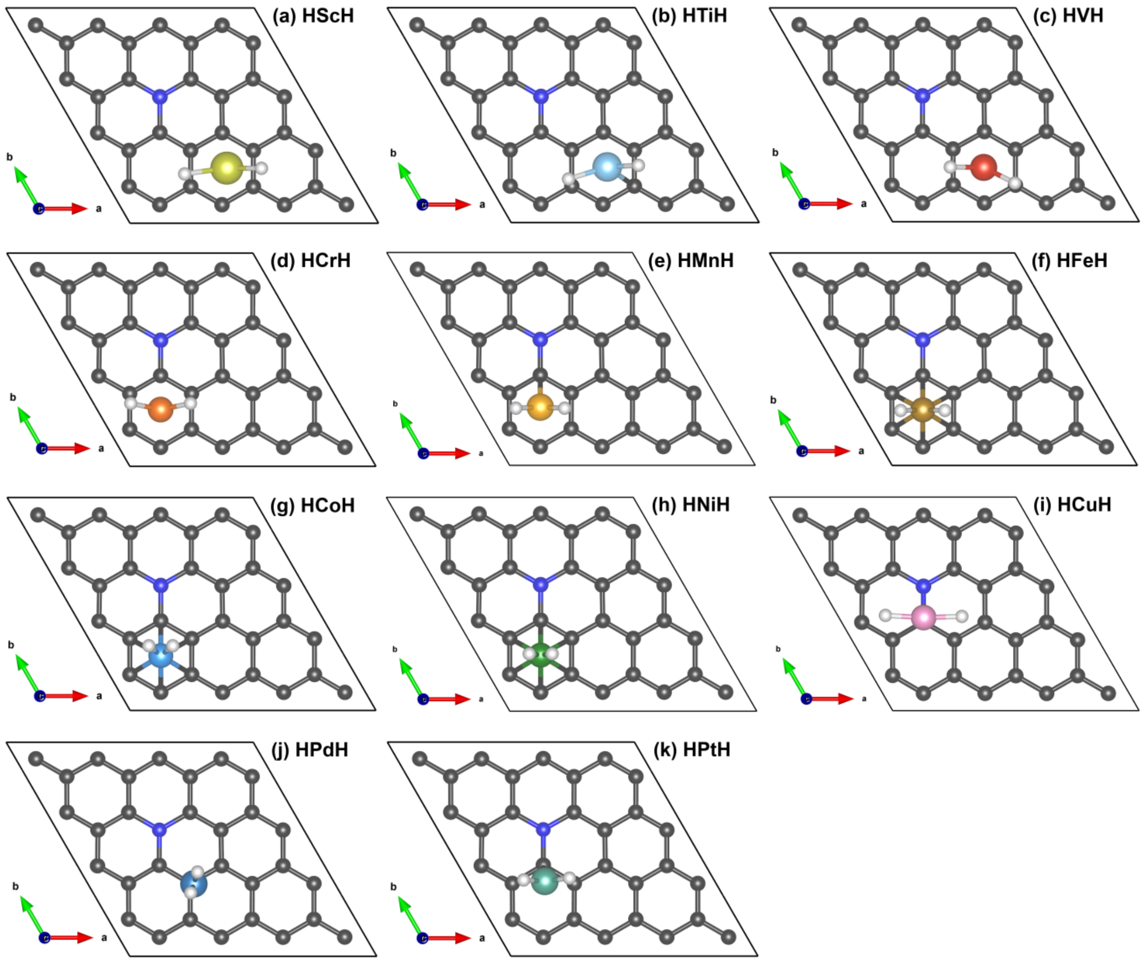


**Figure S13**. Top views of HMH/N-Gr intermediates. (a) HScH/N-Gr, (b) HTiH/N-Gr, (c) HVH/N-Gr, (d) HCrH/N-Gr, (e) HMnH/N-Gr, (f) HFeH/N-Gr, (g) HCoH/N-Gr, (h) HNiH/N-Gr, (i) HCuH/N-Gr, (j) HPdH/N-Gr, and (k) HPtH/N-Gr.

## Transition metal atoms replacing a C-atom of N-Gr (M@N-Gr)

We considered N-Gr as support, and the transition metal atoms M have been incorporated in the lattice replacing a C atom (which corresponds to adsorbing the metal atom on a C-vacancy of the support), Figure S14. All metal atoms from Sc to Cu, Pd and Pt, have been considered. In Table S15 we report the distance between C and each metal, M, and the charge transfer (CT) from M to N-Gr as measured by the QTAIM charge (in |e|).

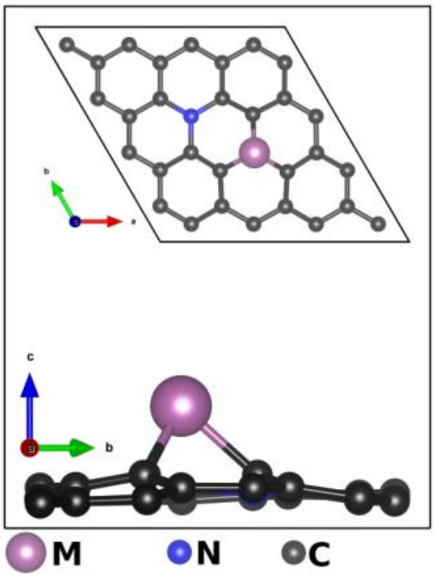


**Figure S14**. Top and side view of M@N-Gr, where M = Sc, Ti, V, Cr, Mn, Fe, Co, Ni, Cu, Pd, and Pt.

**Table S11**. Bond distance (*d*, in Å), and charge transfer (CT, in |e|) from M atoms replacing a C-atom in N-Gr.

| | $d_{C\text{-}M}$ | CT |
|---|---|---|
| Sc@N-Gr | 2.05-2.07 | 1.29 |
| Ti@N-Gr | 1.89-1.97 | 1.31 |
| V@N-Gr | 1.89-1.91 | 1.09 |
| Cr@N-Gr | 1.86-1.88 | 0.94 |
| Mn@N-Gr | 1.81-1.82 | 0.87 |
| Fe@N-Gr | 1.77 | 0.64 |
| Co@N-Gr | 1.76 | 0.50 |
| Ni@N-Gr | 1.79-1.80 | 0.48 |
| Cu@N-Gr | 1.86-1.87 | 0.53 |
| Pd@N-Gr | 1.95-1.96 | 0.29 |
| Pt@N-Gr | 1.93-1.95 | 0.22 |

## Hydrogen adsorption on M@N-Gr

**Table S12**. Adsorption energy ($\Delta E_H$, in eV), Zero-Point-Energy contribution ($\Delta E_{ZPE}$, in eV), Entropy ($T\Delta S_H$, in eV), Gibbs Free Energy ($\Delta G_H$, in eV), bond distances (*d*, in Å), and bond angle (∠HMH °) for one or two H atoms adsorbed on top of M@N-Gr (M = Sc, Ti, V, Cr, Mn, Fe, Co, Ni, Cu, Pd, and Pt). $\Delta E_H$ is the adsorption energy per H atom given with respect to ½ $H_2$; in square bracket [] is reported the energy of M + $H_2$ → HMH.

| | $\Delta E_H$ | $\Delta E_{ZPE}$ | $T\Delta S_H$ | $\Delta G_H$ | $d_{C\text{-}M}$ | $d_{M\text{-}H}$ | $d_{H\text{-}H}$ | ∠H-M-H |
|---|---|---|---|---|---|---|---|---|
| $H_2$ | --- | --- | --- | --- | --- | --- | 0.75 | --- |
| ScH@N-Gr | --- | --- | -0.19 | --- | --- | --- | --- | --- |
| HScH@N-Gr | --- | --- | -0.38 | --- | --- | --- | --- | --- |
| TiH@N-Gr | -0.34 | 0.020 | -0.19 | -0.13 | 1.97-2.00 | 1.79 | --- | --- |
| HTiH@N-Gr | 0.06 [-0.28] | 0.151 | -0.38 | 0.38 [0.25] | 1.90-1.98 | ~2.23 | 0.77 | 20 |

| | | | | | | | | |
|---|---|---|---|---|---|---|---|---|
| VH@N-Gr | -0.31 | 0.036 | -0.19 | -0.08 | 1.89-1.93 | 1.72 | --- | --- |
| HVH@N-Gr | 0.25 [-0.06] | 0.099 | -0.38 | 0.50 [0.42] | 1.88-2.01 | 1.62-1.69 | 1.77 | 64 |
| CrH@N-Gr | -0.40 | 0.036 | -0.19 | -0.17 | ~1.88 | 1.67 | --- | --- |
| HCrH@N-Gr | 0.33 [-0.07] | 0.145 | -0.38 | 0.63 [0.46] | 1.84-1.87 | 1.74-1.81 | 0.88 | 28 |
| MnH@N-Gr | -0.34 | 0.099 | -0.19 | -0.05 | 1.80-1.82 | 1.61 | --- | --- |
| HMnH@N-Gr | -0.02 [-0.36] | 0.143 | -0.38 | 0.21 [0.16] | ~1.85 | 1.80 | 0.83 | 26 |
| FeH@N-Gr | -0.44 | 0.052 | -0.19 | -0.20 | 1.76 | 1.53 | --- | --- |
| HFeH@N-Gr | 0.06 [-0.38] | 0.153 | -0.38 | 0.35 [0.15] | 1.80 | ~1.78 | 0.82 | 27 |
| CoH@N-Gr | 0.16 | 0.026 | -0.19 | 0.38 | 1.76-1.79 | 1.57 | --- | --- |
| HCoH@N-Gr | 0.70 [0.86] | 0.101 | -0.38 | 0.96 [1.34] | 1.76-1.79 | 1.55 | 1.93 | 77 |
| NiH@N-Gr | -0.20 | 0.046 | -0.19 | 0.04 | 1.81-1.83 | 1.54 | --- | --- |
| HNiH@N-Gr | 0.38 [0.18] | 0.102 | -0.38 | 0.62 [0.66] | ~1.85 | ~1.51 | 1.80 | 73 |
| CuH@N-Gr | -0.09 | 0.040 | -0.19 | 0.14 | 1.88-1.94 | 1.52 | --- | --- |
| HCuH@N-Gr | -0.06 [-0.15] | 0.259 | -0.38 | 0.35 [0.49] | 1.82-1.90 | 1.68 | 2.72 | 108 |
| PdH@N-Gr | -0.33 | 0.042 | -0.19 | -0.10 | 1.96-1.99 | 1.68 | --- | --- |
| HPdH@N-Gr | 0.49 [0.16] | 0.105 | -0.38 | 0.75 [0.65] | 1.95-2.03 | 1.63 | 2.10 | 80 |
| PtH@N-Gr | -0.88 | 0.046 | -0.19 | -0.64 | 1.96-2.00 | 1.68 | --- | --- |
| HPtH@N-Gr | -0.01 [-0.89] | 0.132 | -0.38 | 0.26 [-0.38] | 1.96-2.02 | 1.65 | 2.14 | 81 |

The adsorption of the second hydrogen atoms leads always to a dihydrogen or dihydride complex, with the only exception of Sc@N-Gr, where we did not observe the formation of any complex. In this case, one must use the original approach based on a single intermediate to describe the HER process.

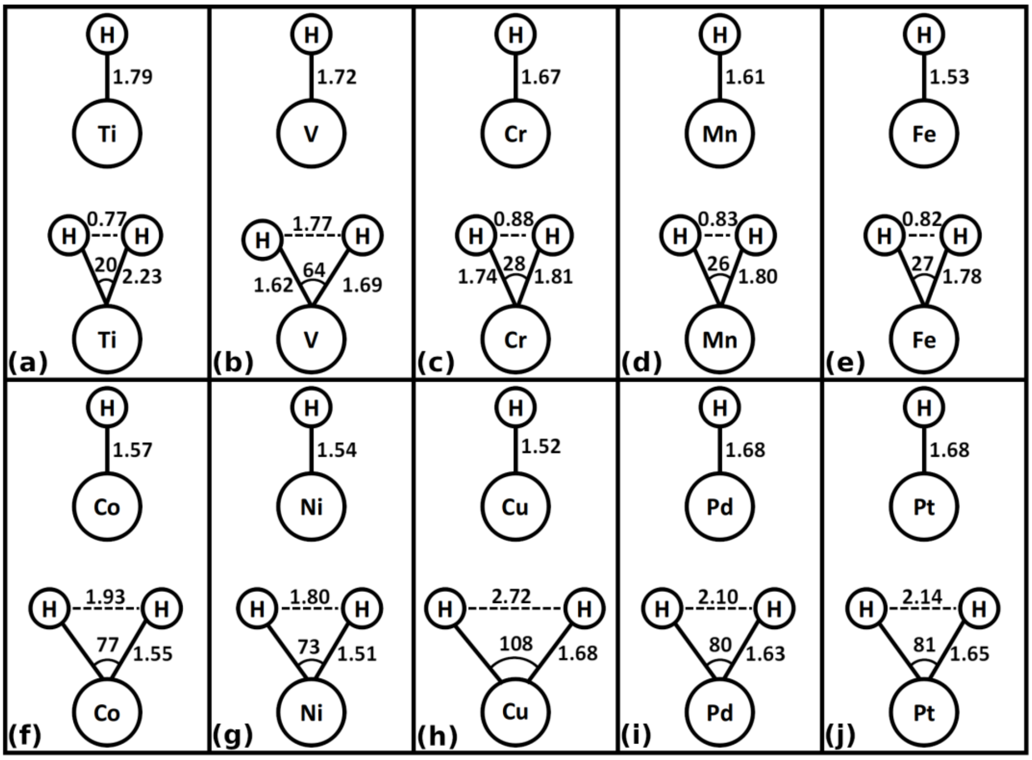


**Figure S15.** Schematic view of the MH@N-Gr and HMH@N-Gr intermediates. Bond lengths are reported in Å, bond angles in degrees (º). (a) TiH@N-Gr, HTiH@N-Gr; (b) VH@N-Gr, HVH@N-Gr; (c) CrH@N-Gr, HCrH@N-Gr; (d) MnH@N-Gr, HMnH@N-Gr; (e) FeH@N-Gr, HFeH@N-Gr; (f)

CoH@N-Gr, HCoH@N-Gr; (g) NiH@N-Gr, HNiH@N-Gr; (h) CuH@N-Gr, HCuH@N-Gr; (i) PdH@N-Gr, HPdH@N-Gr; and (j) PtH@N-Gr, HPtH@N-Gr.

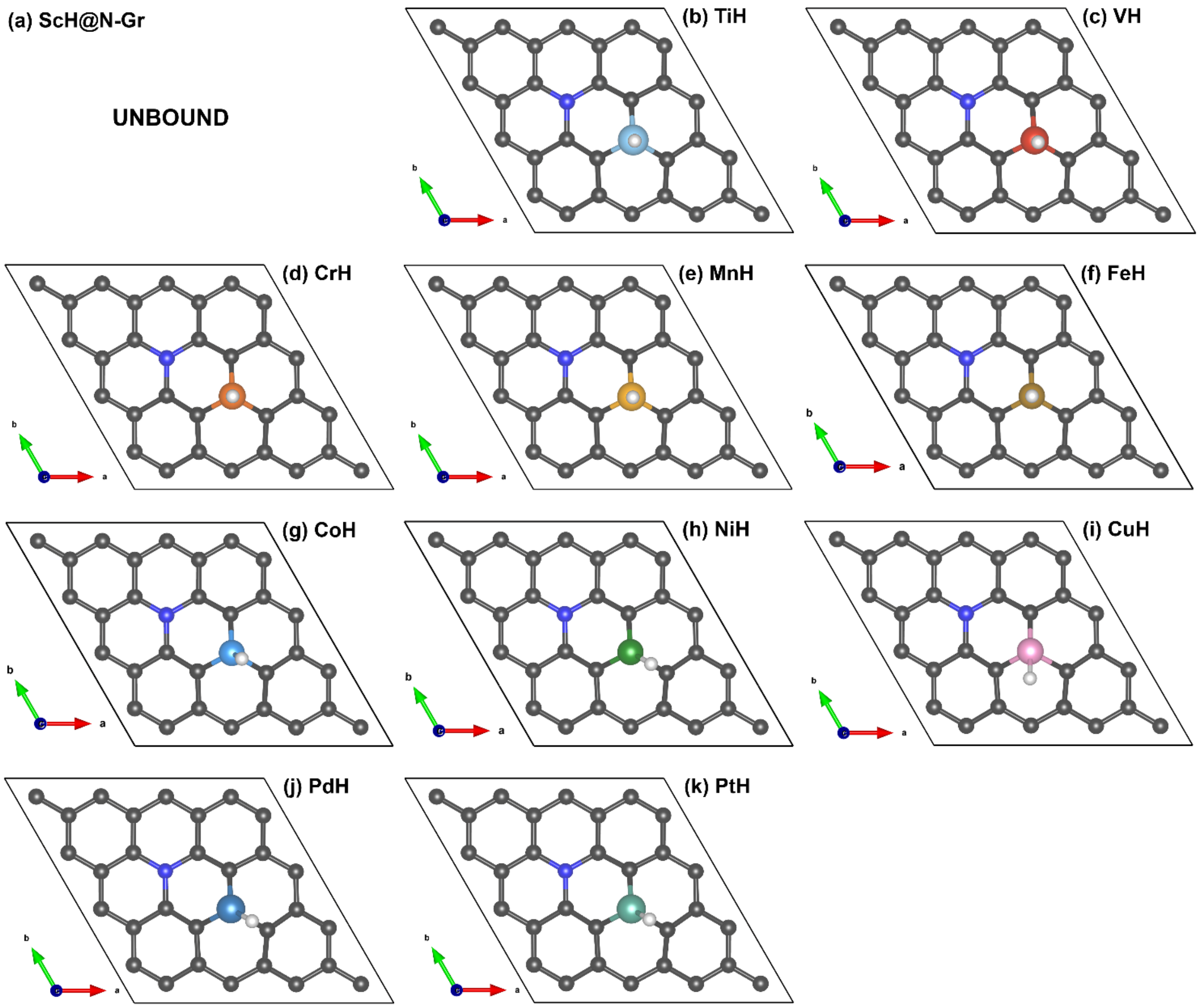


**Figure S16**. Top views of the first H atom adsorbed on M@N-Gr. (a) ScH@N-Gr, (b) TiH@N-Gr, (c) VH@N-Gr, (d) CrH@N-Gr, (e) MnH@N-Gr, (f) FeH@N-Gr, (g) CoH@N-Gr, (h) NiH@N-Gr, (i) CuH@N-Gr, (j) PdH@N-Gr, and (k) PtH@N-Gr.

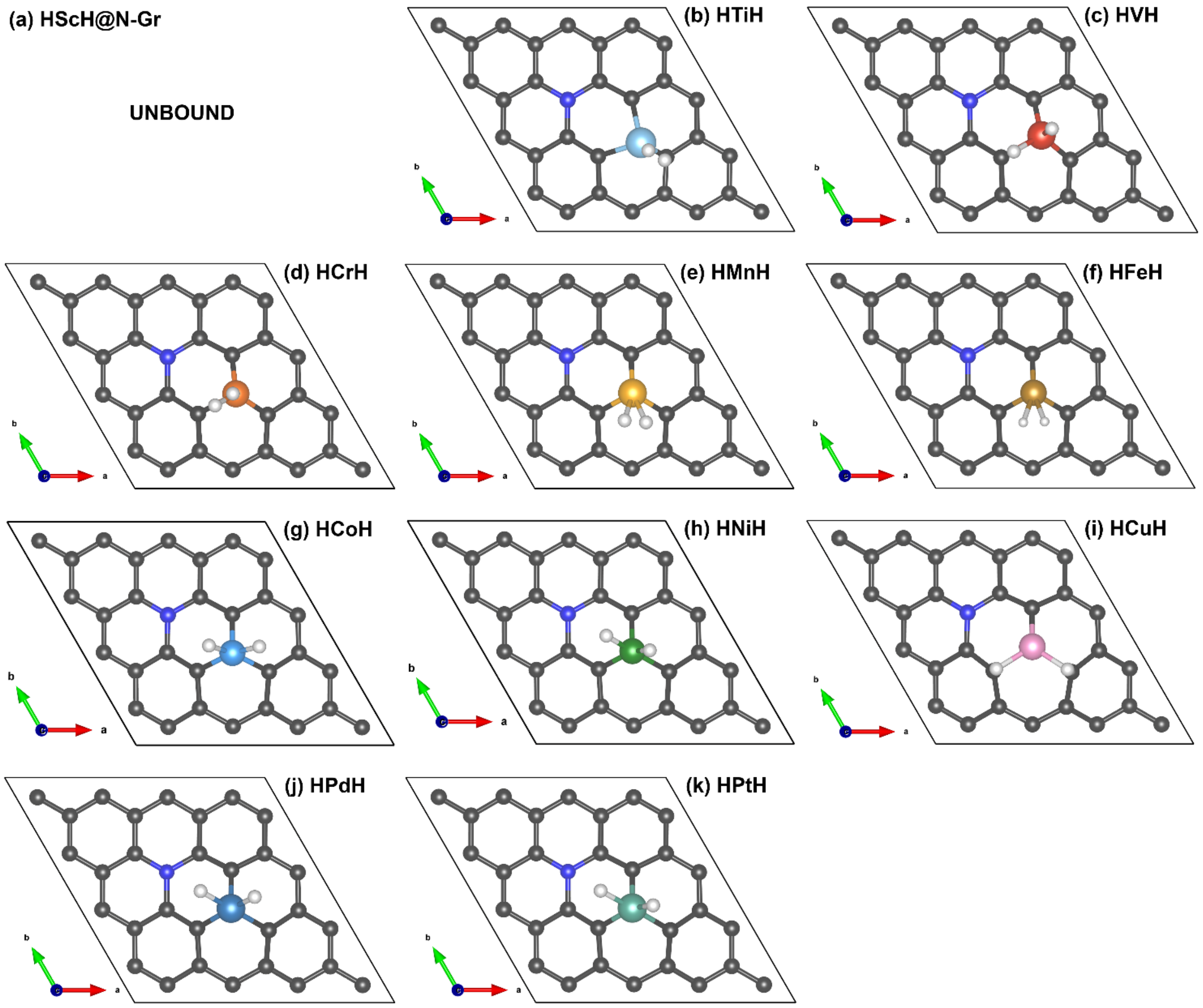


**Figure S17**. Top views of HMH@N-Gr intermediates. (a) HScH@N-Gr, (b) HTiH@N-Gr, (c) HVH@N-Gr, (d) HCrH@N-Gr, (e) HMnH@N-Gr, (f) HFeH@N-Gr, (g) HCoH@N-Gr, (h) HNiH@N-Gr, (i) HCuH@N-Gr, (j) HPdH@N-Gr, and (k) HPtH@N-Gr.

## Transition metal atoms replacing a C atom of 3N-Gr (M@3N-Gr)

In order to generate the M@3N-Gr models of SACs, we first doped the pristine graphene with 3 N atoms, and we considered the metal atom M incorporated in a C-vacancy with the three adjacent N atoms (Figure S18). In Table S13 we report the N-M bond distance and the charge transfer from M to 3N-Gr as computed with the QTAIM analysis.

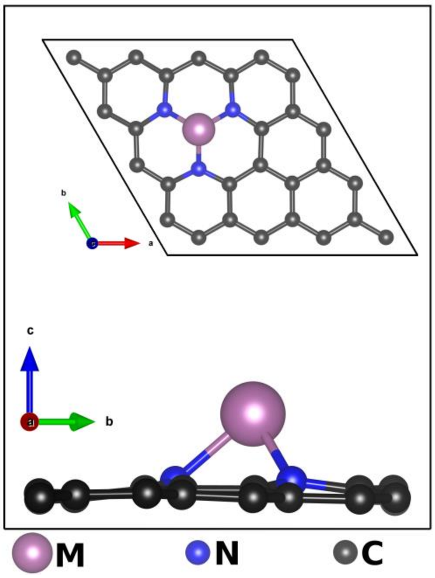


**Figure S18.** Top and side views of M@3N-Gr, where M = Sc, Ti, V, Cr, Mn, Fe, Co, Ni, Cu, Pd, and Pt.

**Table S13.** Bond distance (*d*, in Å), and charge transfer (CT, in |e|) from M to the 3N-Gr.

| | $d_{\text{N-M}}$ | CT |
|---|---|---|
| Sc@3N-Gr | 2.02 | 1.25 |
| Ti@3N-Gr | 1.94 | 1.23 |
| V@3N-Gr | 1.90 | 1.14 |
| Cr@3N-Gr | 1.92 | 1.09 |
| Mn@3N-Gr | 1.67 | 0.78 |
| Fe@3N-Gr | 1.86 | 0.95 |
| Co@3N-Gr | 1.82 | 0.78 |
| Ni@3N-Gr | 1.85 | 0.76 |
| Cu@3N-Gr | 1.93 | 0.50 |
| Pd@3N-Gr | 2.16 | 0.45 |
| Pt@3N-Gr | 2.18 | 0.41 |

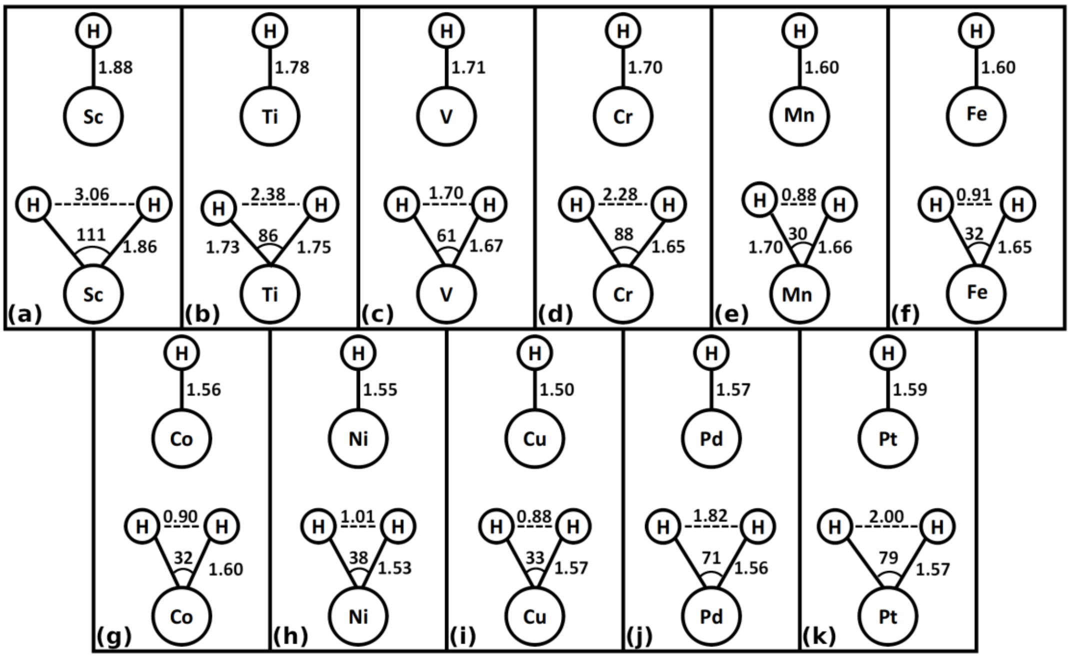


**Figure S19**. Schematic view of the MH@3N-Gr and HMH@3N-Gr intermediates. The bond lengths and angles are reported in Å and degrees (º). (a) HSc@3N-Gr, HScH@3N-Gr; (b) TiH@3N-Gr, HTiH@3N-Gr; (c) VH@3N-Gr, HVH@3N-Gr; (d) CrH@3N-Gr, HCrH@3N-Gr; (e) MnH@3N-Gr, HMnH@3N-Gr; (f) FeH@3N-Gr, HFeH@3N-Gr; (g) CoH@3N-Gr, HCoH@3N-Gr; (h) NiH@3N-Gr, HNiH@3N-Gr; (i) CuH@3N-Gr, HCuH@3N-Gr; (j) PdH@3N-Gr, HPdH@3N-Gr; and (k) PtH@3N-Gr, HPtH@3N-Gr.

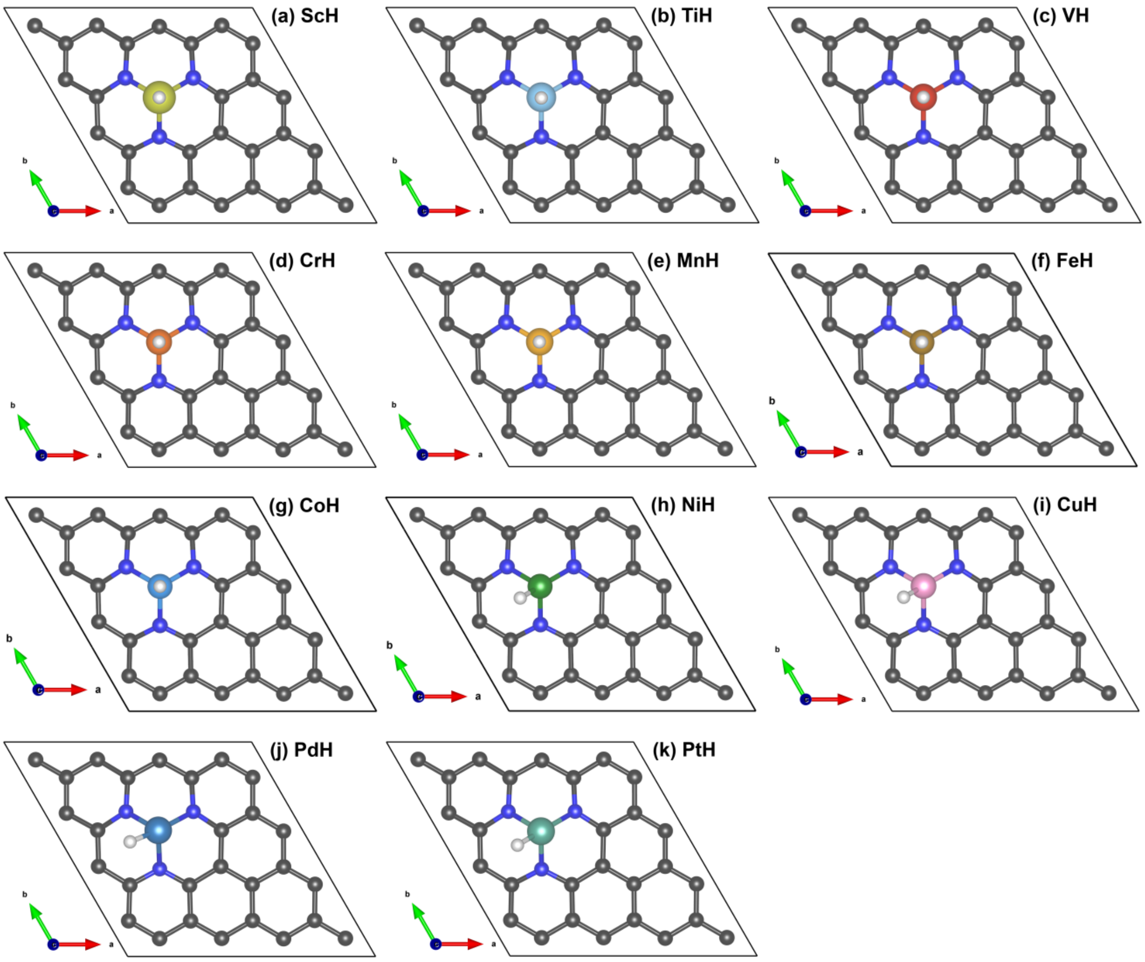


**Figure S20**. Top views of the first H adsorbed on M@3N-Gr. (a) ScH@3N-Gr, (b) TiH@3N-Gr, (c) VH@3N-Gr, (d) CrH@3N-Gr, (e) Mn@3N-Gr, (f) FeH@3N-Gr, (g) CoH@3N-Gr, (h) NiH@3N-Gr, (i) CuH@3N-Gr, (j) PdH@3N-Gr, and (k) PtH@3N-Gr.

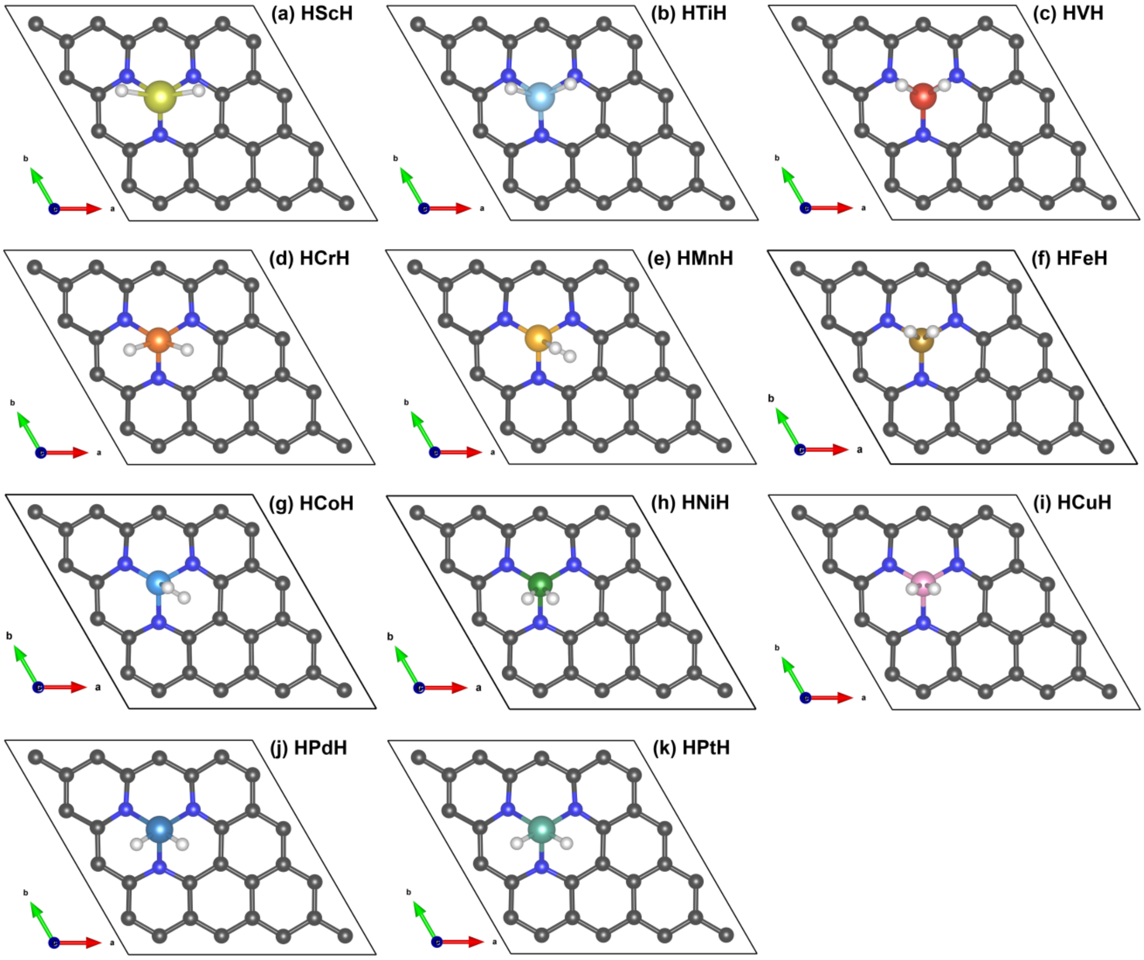


**Figure S21**. Top views of HMH@3N-Gr intermediates. (a) HScH@3N-Gr, (b) HTiH@3N-Gr, (c) HVH@3N-Gr, (d) HCrH@3N-Gr, (e) HMn@3N-Gr, (f) HFeH@3N-Gr, (g) HCoH@3N-Gr, (h) HNiH@3N-Gr, (i) HCuH@3N-Gr, (j) HPdH@3N-Gr, and (k) HPtH@3N-Gr.

## Transition metal atoms adsorbed on $MoS_2$ ($M/MoS_2$)

We considered transition metal atoms adsorbed on a $MoS_2$ monolayer. We adsorb the M atom on a hollow site where M is coordinated by three S atoms. In Table S14 we report the M-S bond distance (in Å) and the charge transfer from M to $MoS_2$ as measured by the QTAIM analysis. The binding energy was computed as:

$$BE = M/MoS_2 - (MoS_2 + M)$$

**Table S14**. Binding energy (BE, in eV), bond distance (*d*, in Å) and charge transfer (CT, in |e|) from M atoms to $MoS_2$.

| | BE | $d_{S\text{-}M}$ | CT |
|---|---|---|---|
| Sc/$MoS_2$ | -2.92 | 2.31 | 1.34 |
| Ti/$MoS_2$ | -3.23 | 2.21 | 1.24 |
| V/$MoS_2$ | -2.25 | 2.15 | 1.06 |
| Cr/$MoS_2$ | -1.35 | 2.21 | 0.91 |

| | | | |
|---|---|---|---|
| Mn/$MoS_2$ | -1.60 | 2.14 | 0.82 |
| Fe/$MoS_2$ | -2.49 | 2.09 | 0.63 |
| Co/$MoS_2$ | -2.93 | 2.06 | 0.44 |
| Ni/$MoS_2$ | -3.46 | 2.06 | 0.34 |
| Cu/$MoS_2$ | -1.55 | 2.30 | 0.40 |
| Pd/$MoS_2$ | -2.26 | 2.39 | 0.19 |
| Pt/$MoS_2$ | -2.72 | 2.33 | 0.09 |

## Hydrogen adsorption on M/$MoS_2$

**Table S15**. Adsorption energy ($\Delta E_H$, in eV), Zero-Point-Energy contribution ($\Delta E_{ZPE}$, in eV), Entropy ($T\Delta S_H$, in eV), Gibbs Free Energy ($\Delta G_H$, in eV), bond distances ($d$, in Å), and bond angle (∠HMH °) for one or two H atoms adsorbed on top of M/$MoS_2$ (M = Sc, Ti, V, Cr, Mn, Fe, Co, Ni, Cu, Pd, and Pt). $\Delta E_H$ is the adsorption energy per H atom given with respect to ½ $H_2$; in square bracket [] is reported the energy of M + $H_2 \rightarrow$ HMH.

| | $\Delta E_H$ | $\Delta E_{ZPE}$ | $T\Delta S_H$ | $\Delta G_H$ | $d_{S\text{-}M}$ | $d_{M\text{-}H}$ | $d_{H\text{-}H}$ | ∠H-M-H |
|---|---|---|---|---|---|---|---|---|
| $H_2$ | --- | --- | --- | --- | --- | --- | 0.75 | --- |
| ScH/$MoS_2$ | -0.22 | -0.011 | -0.19 | -0.04 | 2.41 | 1.82 | --- | --- |
| HScH/$MoS_2$ | 0.14 [-0.08] | -0.001 | -0.38 | 0.34 [0.30] | 2.54-2.67 | 1.81 | 2.91 | 107 |
| TiH/$MoS_2$ | -0.30 | 0.003 | -0.19 | -0.11 | 2.25 | 1.73 | --- | --- |
| HTiH/$MoS_2$ | -0.32 [-0.62] | 0.113 | -0.38 | -0.02 [-0.13] | 2.21 | 2.00 | 0.80 | 23 |
| VH/$MoS_2$ | -0.29 | 0.031 | -0.19 | -0.07 | 2.16 | 1.65 | --- | --- |
| HVH/$MoS_2$ | -0.36 [-0.65] | 0.100 | -0.38 | -0.10 [-0.17] | 2.18 | 1.91 | 0.82 | 25 |
| CrH/$MoS_2$ | -0.25 | 0.001 | -0.19 | -0.06 | 2.35 | 1.65 | --- | --- |
| HCrH/$MoS_2$ | -0.22 [-0.47] | 0.107 | -0.38 | 0.08 [0.02] | 2.20-2.29 | 1.86 | 0.83 | 25 |
| MnH/$MoS_2$ | -0.03 | -0.007 | -0.19 | 0.15 | 2.28 | 1.62 | --- | --- |
| HMnH/$MoS_2$ | -0.48[-0.51] | 0.157 | -0.38 | -0.12 [0.03] | 2.17 | 1.82 | 0.82 | 26 |
| FeH/$MoS_2$ | 0.03 | 0.026 | -0.19 | 0.25 | 2.16 | 1.57 | --- | --- |
| HFeH/$MoS_2$ | -0.41 [-0.38] | 0.117 | -0.38 | -0.13 [0.12] | 2.12-2.15 | 1.68 | 0.85 | 29 |
| CoH/$MoS_2$ | 0.04 | 0.035 | -0.19 | 0.27 | 2.05 | 1.46 | --- | --- |
| HCoH/$MoS_2$ | -0.67 [-0.63] | 0.127 | -0.38 | -0.39 [-0.12] | 2.09 | 1.64 | 0.85 | 30 |
| NiH/$MoS_2$ | 0.52 | 0.013 | -0.19 | 0.72 | 2.12 | 1.49 | --- | --- |
| HNiH/$MoS_2$ | -0.99 [-0.47] | 0.115 | -0.38 | -0.69 [0.03] | 2.13 | 1.68 | 0.82 | 28 |
| CuH/$MoS_2$ | -0.26 | 0.015 | -0.19 | -0.06 | 2.39 | 1.51 | --- | --- |
| HCuH/$MoS_2$ | -0.06 [-0.32] | 0.115 | -0.38 | 0.24 [0.18] | 2.34 | 1.73 | 0.80 | 27 |
| PdH/$MoS_2$ | 0.69 | 0.013 | -0.19 | 0.89 | 2.46 | 1.59 | --- | --- |
| HPdH/$MoS_2$ | -1.27 [-0.58] | 0.114 | -0.38 | -0.98 [-0.09] | 2.37 | 1.80 | 0.83 | 27 |
| PtH/$MoS_2$ | -0.20 | 0.036 | -0.19 | 0.03 | 2.36 | 1.58 | --- | --- |
| HPtH/$MoS_2$ * | -1.66 [-1.86] | 0.144 | -0.38 | -1.37 [-1.34] | 2.44 | 1.56 | 2.06 | 83 |

* When we adsorb the second H on PtH/$MoS_2$, the Pt atom moves from the hollow site to a bridge site.

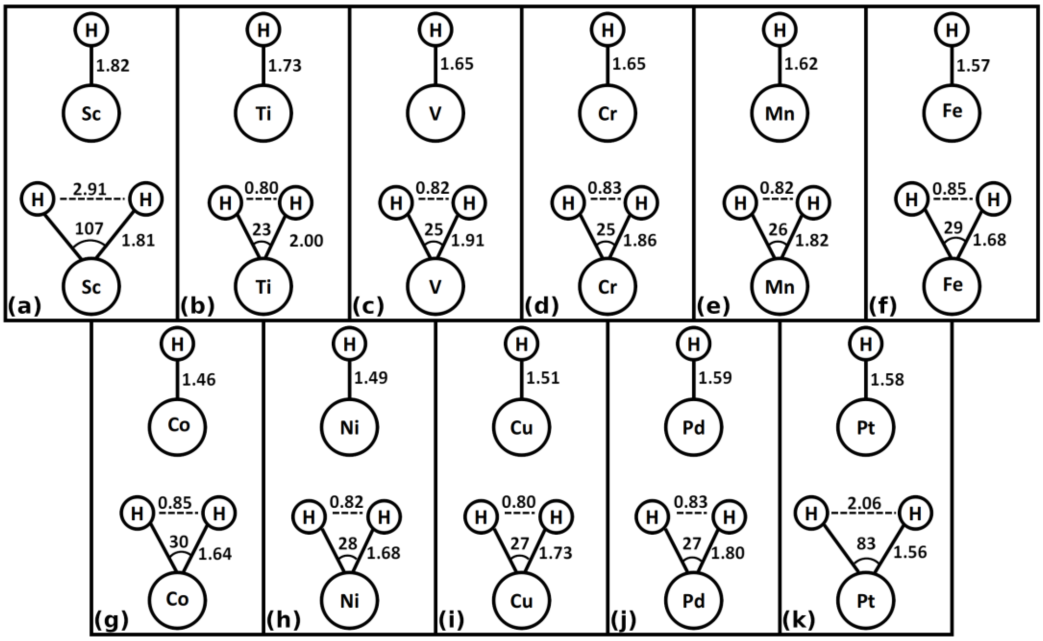


**Figure S22**. Schematic view of the $MH/MoS_2$ and $HMH/MoS_2$ intermediates. The bond lengths and angles are reported in Å and degrees (°), respectively. (a) $HSc/MoS_2$, $HScH/MoS_2$; (b) $TiH/MoS_2$, $HTiH/MoS_2$; (c) $VH/MoS_2$, $HVH/MoS_2$; (d) $CrH/MoS_2$, $HCrH/MoS_2$; (e) $MnH/MoS_2$, $HMnH/MoS_2$; (f) $FeH/MoS_2$, $HFeH/MoS_2$; (g) $CoH/MoS_2$, $HCoH/MoS_2$; (h) $NiH/MoS_2$, $HNiH/MoS_2$; (i) $CuH/MoS_2$, $HCuH/MoS_2$; (j) $PdH/MoS_2$, $HPdH/MoS_2$; and (k) $PtH/MoS_2$, $HPtH/MoS_2$.

## $PtH/MoS_2$ and $HPtH/MoS_2$ complexes

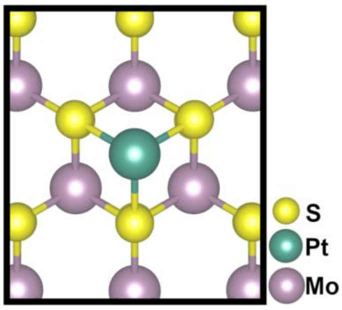


**Figure S23**. Top view of Pt adsorbed on the hollow site; BE(Pt)=-2.72 eV.

**Table S16.** Adsorption energy ($\Delta E_H$, in eV), Zero-Point-Energy contribution ($\Delta E_{ZPE}$, in eV), Entropy ($T\Delta S_H$, in eV), and Gibbs Free Energy ($\Delta G_H$, in eV) for the $PtH/MoS_2$ and $HPtH/MoS_2$ intermediates in the hollow site.

| | $\Delta E_H$ | $\Delta E_{ZPE}$ | $T\Delta S_H$ | $\Delta G_H$ |
|---|---|---|---|---|
| $PtH/MoS_2$ | -0.20 | 0.04 | -0.19 | 0.03 |
| $HPtH/MoS_2$ | -1.66 | 0.10 | -0.19 | -1.37 |
| total | -1.86 | 0.14 | -0.38 | -1.34 |

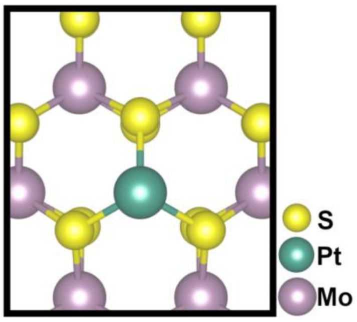


**Figure S24**. Top view of of Pt adsorbed on the Mo top site; BE(Pt) = -3.34 eV

**Table S17**. Adsorption energy ($\Delta E_H$, in eV), Zero-Point-Energy contribution ($\Delta E_{ZPE}$, in eV), Entropy ($T\Delta S_H$, in eV), and Gibbs Free Energy ($\Delta G_H$, in eV) for the $PtH/MoS_2$ and $HPtH/MoS_2$ intermediates in the Mo top site.

| | $\Delta E_H$ | $\Delta E_{ZPE}$ | $T\Delta S_H$ | $\Delta G_H$ |
|---|---|---|---|---|
| $PtH/MoS_2$ | -0.27 | 0.04 | -0.19 | -0.04 |
| $HPtH/MoS_2$ | -1.03 | 0.10 | -0.19 | -0.74 |
| total | -1.30 | 0.14 | -0.38 | -0.78 |

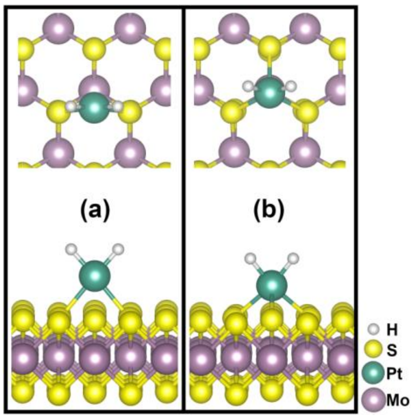


**Figure S25**. Side and top views of $HPtH/MoS_2$ intermediates on (a) hollow and (b) Mo top sites.

## Vibrational frequencies of HMH complexes

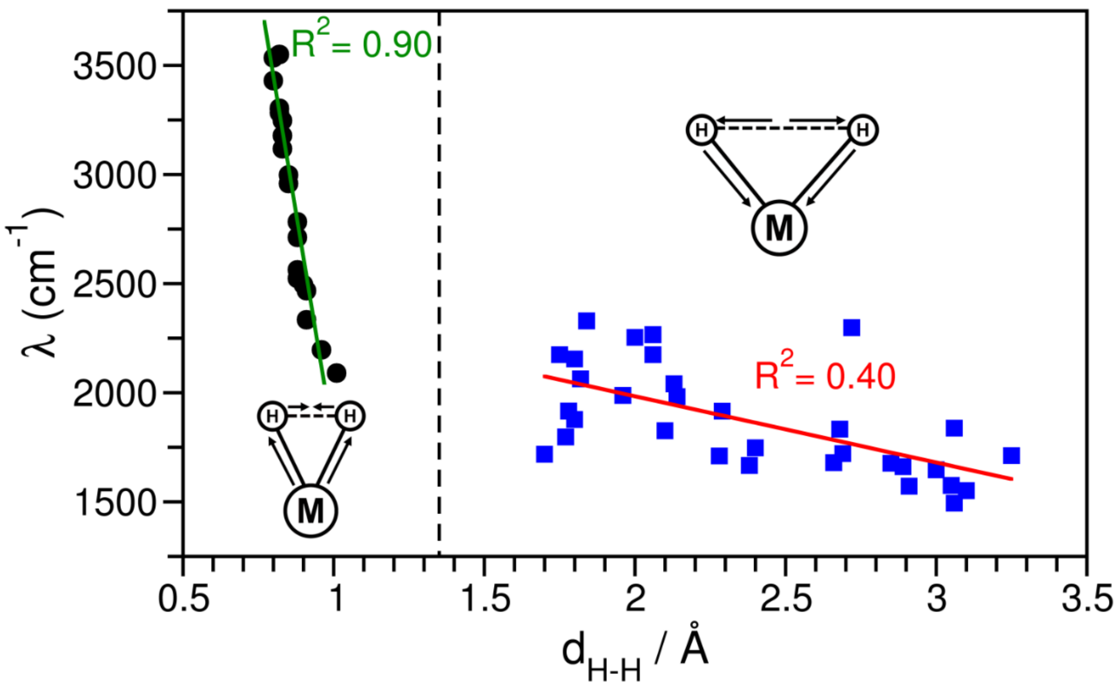


**Figure S26**. Correlation between the vibrational frequency (H-H, or M-H), and the H-H distance for the various HMH complexes considered in this work. The distinct feature of dihydrogen (left) and dihydride (right) complexes provides a fingerprint of the nature of the HMH complex.

## Comparison of kinetics based on one or two intermediates

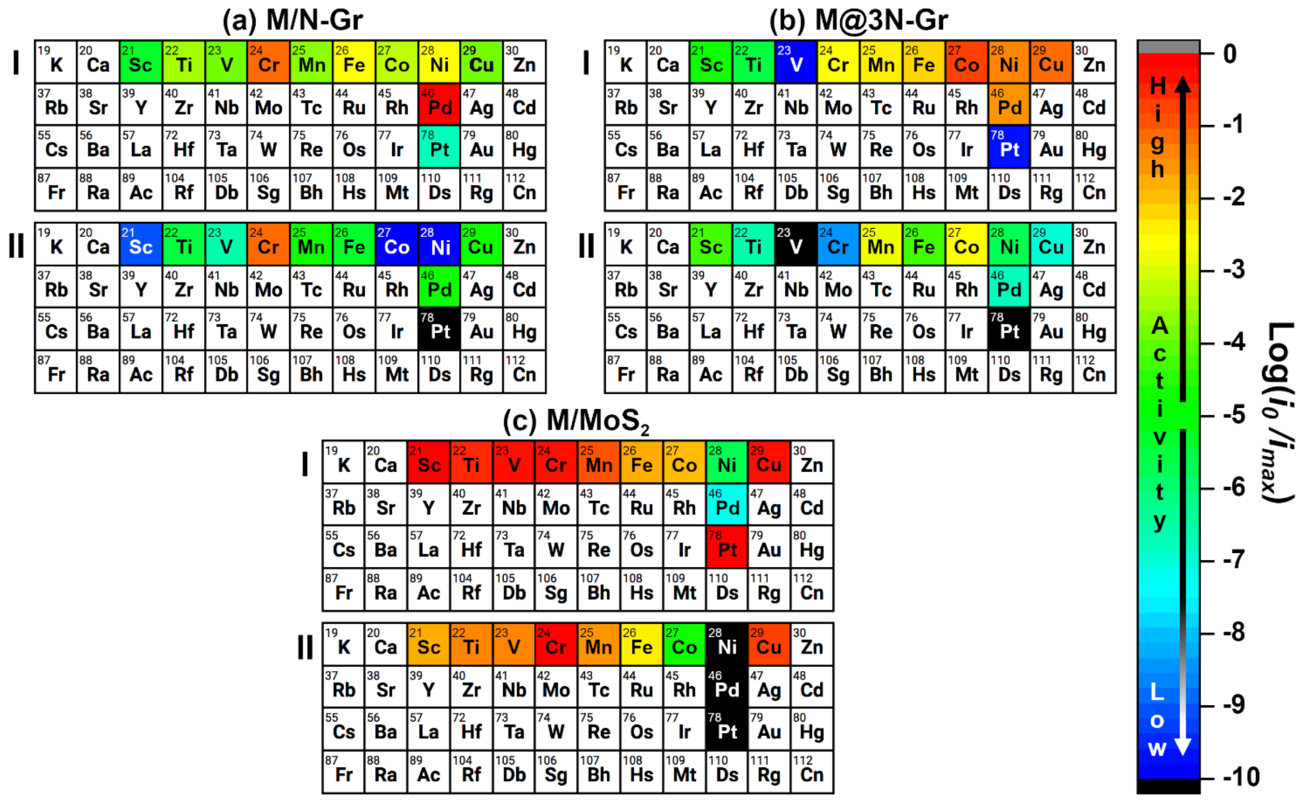

**Figure S27**. Predicted efficiency for the HER reaction for the series of (a) M/N-Gr, (b) M@3N-Gr, and (c) M/$MoS_2$. The color refers to the ratio of the exchange current $i_0$ for a given atom and the maximum exchange current, $i_{max}$, and provides a visual measure of the expected HER activity. (I) classical model based on a single MH intermediate; (II) model based on both MH and HMH intermediates (two-hydrogens complex). The best catalysts (in red) have Log($i_0/i_{max}$) close to zero; the worse catalysts (in blue) have Log($i_0/i_{max}$) close to -10. Atoms are coloured in black when Log($i_0/i_{max}$) < -10.

## Solvent effects

The implicit solvent effects have been introduced as a patch to the original VASP code, which was developed and implemented by Hennig and Arias.[20]

**Table S18 -** Effect of the implicit solvent on the adsorption energies of the first and second hydrogen on M@4N-Gr (M=Co, W, and Ni) with the PBE functional. The HNiH@4N-Gr complex does not form but we are reporting the value for the MH complex for comparative purposes.

| Metal | Solvent | $\Delta E^{(1)}$ / eV | $\Delta E^{(2)}$ / eV | $\Delta E^{(3)}$ / eV |
|---|---|---|---|---|
| Co@4N-Gr | No | -0.11 | 0.12 | 0.01 |
| | YES | -0.13 | 0.09 | -0.04 |
| | | | | |
| W@4N-Gr | No | -1.12 | -0.25 | -1.37 |
| | YES | -1.16 | -0.29 | -1.45 |
| | | | | |
| Ni@4N-Gr | No | 1.41 | -1.46 | -0.05 |
| | YES | 1.37 | -1.42 | -0.05 |

## References


(1) Mefford, J. T.; Zhao, Z.; Bajdich, M.; Chueh, W. C. Interpreting Tafel Behavior of Consecutive Electrochemical Reactions through Combined Thermodynamic and Steady State Microkinetic Approaches. *Energy Environ. Sci.* **2020**, *13* (2), 622–634.

(2) Koper, M. T. M. Activity Volcanoes for the Electrocatalysis of Homolytic and Heterolytic Hydrogen Evolution. *J. Solid State Electrochem.* **2016**, *20* (4), 895–899.

(3) Kresse, G.; Hafner, J. Ab Initio Molecular Dynamics for Liquid Metals. *Phys. Rev. B* **1993**, *47* (1), 558–561.

(4) Kresse, G.; Hafner, J. Ab Initio Molecular-Dynamics Simulation of the Liquid-Metal–Amorphous-Semiconductor Transition in Germanium. *Phys. Rev. B* **1994**, *49* (20), 14251–14269.

(5) Kresse, G.; Furthmüller, J. Efficiency of Ab-Initio Total Energy Calculations for Metals and Semiconductors Using a Plane-Wave Basis Set. *Comput. Mater. Sci.* **1996**, *6* (1), 15–50.

(6) Perdew, J. P.; Burke, K.; Ernzerhof, M. Generalized Gradient Approximation Made Simple. *Phys. Rev. Lett.* **1996**, *77* (18), 3865–3868.

(7) Grimme, S.; Antony, J.; Ehrlich, S.; Krieg, H. A Consistent and Accurate Ab Initio Parametrization of Density Functional Dispersion Correction (DFT-D) for the 94 Elements H-

Pu. *J. Chem. Phys.* **2010**, *132* (15), 154104.

(8) Blöchl, P. E. Projector Augmented-Wave Method. *Phys. Rev. B* **1994**, *50* (24), 17953–17979.

(9) Kresse, G.; Joubert, D. From Ultrasoft Pseudopotentials to the Projector Augmented-Wave Method. *Phys. Rev. B* **1999**, *59* (3), 1758–1775.

(10) Monkhorst, H. J.; Pack, J. D. Special Points for Brillouin-Zone Integrations. *Phys. Rev. B* **1976**, *13* (12), 5188–5192.

(11) Bader, R. F. W. Atoms in Molecules. *Acc. Chem. Res.* **1985**, No. 18, 9–15.

(12) Henkelman, G.; Arnaldsson, A.; Jónsson, H. A Fast and Robust Algorithm for Bader Decomposition of Charge Density. *Comput. Mater. Sci.* **2006**, *36* (3), 354–360.

(13) Sanville, E.; Kenny, S. D.; Smith, R.; Henkelman, G. Improved Grid-Based Algorithm for Bader Charge Allocation. *J. Comput. Chem.* **2007**, *28* (5), 899–908.

(14) Tang, W.; Sanville, E.; Henkelman, G. A Grid-Based Bader Analysis Algorithm without Lattice Bias. *J. Phys. Condens. Matter* **2009**, *21* (8), 84204.

(15) Walsh, A.; Sokol, A. A.; Buckeridge, J.; Scanlon, D. O.; Catlow, C. R. A. Oxidation States and Ionicity. *Nat. Mater.* **2018**, *17* (11), 958–964.

(16) Walsh, A.; Sokol, A. A.; Buckeridge, J.; Scanlon, D. O.; Catlow, C. R. A. Electron Counting in Solids: Oxidation States, Partial Charges, and Ionicity. *J. Phys. Chem. Lett.* **2017**, *8* (9), 2074–2075.

(17) Qiao, W.; Xu, W.; Xu, X.; Wu, L.; Yan, S.; Wang, D. Construction of Active Orbital via Single-Atom Cobalt Anchoring on the Surface of 1T-$MoS_2$ Basal Plane toward Efficient Hydrogen Evolution. *ACS Appl. Energy Mater.* **2020**, *3* (3), 2315–2322.

(18) Zhang, L.; Jia, Y.; Gao, G.; Yan, X.; Chen, N.; Chen, J.; Soo, M. T.; Wood, B.; Yang, D.; Du, A.; Yao, X. Graphene Defects Trap Atomic Ni Species for Hydrogen and Oxygen Evolution Reactions. *Chem* **2018**, *4* (2), 285–297.

(19) Hossain, M. D.; Liu, Z.; Zhuang, M.; Yan, X.; Xu, G.-L.; Gadre, C. A.; Tyagi, A.; Abidi, I. H.; Sun, C.-J.; Wong, H.; Guda, A.; Hao, Y.; Pan, X.; Amine, K.; Luo, Z. Rational Design of Graphene-Supported Single Atom Catalysts for Hydrogen Evolution Reaction. *Adv. Energy Mater.* **2019**, *9* (10), 1803689.

(20) Mathew, K.; Sundararaman, R.; Letchworth-Weaver, K.; Arias, T. A.; Hennig, R. G. Implicit Solvation Model for Density-Functional Study of Nanocrystal Surfaces and Reaction Pathways. *J. Chem. Phys.* **2014**, *140* (8), 84106.
